# Lignin Extraction and Condensation as a Function of Temperature, Residence Time, and Solvent System in Flow-through Reactors

**DOI:** 10.1021/acssuschemeng.5c04198

**Published:** 2025-08-01

**Authors:** David G. Brandner, Jaime Gracia Vitoria, Jacob K. Kenny, Jeremy R. Bussard, Jun Hee Jang, Sean P. Woodworth, Karolien Vanbroekhoven, Yuriy Román-Leshkov, Gregg T. Beckham

**Affiliations:** † Renewable Resources and Enabling Sciences Center, 53405 National Renewable Energy Laboratory, Golden, Colorado 80401, United States; ‡ 54520 Flemish Institute for Technological Research (Vito N.V.), Boeretang 200, Mol 2400, Belgium; § Department of Chemical Engineering, 2167 Massachusetts Institute of Technology, Cambridge, Massachusetts 02139, United States

**Keywords:** lignin-first biorefining, lignin valorization, lignin condensation, flow-through reactor, reductive catalytic fractionation

## Abstract

Solvolytic extraction of lignin from biomass is a critical step in lignin-first biorefining, including the reductive catalytic fractionation (RCF) process. Key to optimal RCF processing is the ability to rapidly extract lignin from biomass at high delignification extents and transfer the lignin molecules to a catalyst surface in a time frame that minimizes lignin condensation reactions. Here, we use a flow-through reactor to study the effects of temperature (175–250 °C), residence time (9 to 36 min), and solvent composition (methanol and methanol–water) on lignin extraction and condensation. We evaluated three metrics at each condition: total delignification, delignification rate, and extent of condensation, the latter measured by a decrease in monomer yield for batch hydrogenolysis reactions of solvolysis liquor compared to batch RCF reactions. We observe that delignification is predominantly determined by temperature, while residence time dictates the lignin condensation extent. Moreover, the extent of both extraction and condensation increased in the methanol–water solvent system compared to that in the methanol system. Lignin extracted in methanol is stable up to 18-min residence times at or below 225 °C, while a majority of the lignin extracted in methanol–water is condensed with a 9-min residence time at 200 °C. These results can inform reactor designs and solvent selection for lignin-first biorefining processes that aim to physically separate the biomass and catalyst.

## Graphical abstract

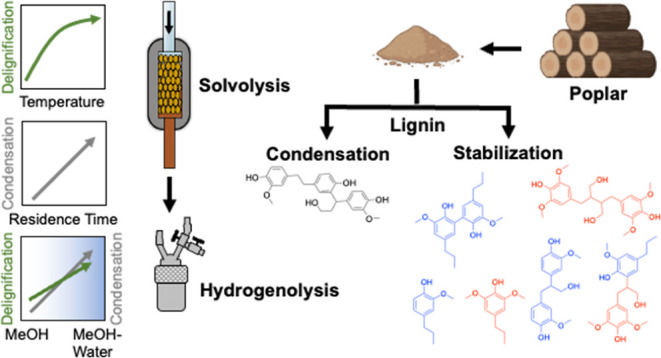

## Introduction

Reductive catalytic fractionation (RCF) is a lignin-first biorefining approach that enables lignin extraction from whole biomass and simultaneous catalytic depolymerization and stabilization of the reactive lignin-derived species, thus preventing condensation reactions to yield stable lignin oils.^1–10^ Many parameters have been studied to enable the RCF process, including feedstock, solvent, catalyst, and reactor configuration.^11–18^ Efforts typically focus on maximizing aromatic monomer yield through the cleavage of aryl-ether bonds, and monomer yields of ∼30–40 wt % are commonly reported for RCF of hardwoods, such as birch and poplar.^15,19^

An important factor dictating the aromatic monomer yield in RCF is the competition between catalytic stabilization and lignin condensation.^2,3,8,9,20–33^ While recent studies have advanced understanding of the process of catalytic stabilization, comparably less is understood regarding the impact of RCF process parameters on lignin condensation.^14,34^ In the presence of added acids, the consensus is that the α-hydroxyl group of the β–O–4 linkage can undergo protonation, leading to dehydration and the formation of a benzylic carbocation, which can form C–C bonds through nucleophilic attack by an aryl unit in another lignin fragment.^9,31,35^ These deleterious condensation reactions can compete with extraction and stabilization reactions to limit the aromatic monomer yield in RCF.^36^ While most lignin-first studies are conducted in stirred batch reactors, various flow-through (FT) reactors have been used to study solvolysis and catalysis as separate reactions.^12,15,16,37–42^ Previously, we demonstrated that FT lignin extraction using methanol at 225 °C produced an uncondensed, native-like lignin due to the short residence time (∼18 min).^15^ These results suggest an opportunity to advance the RCF process by defining the temperature and residence time boundaries for various solvent systems to minimize detrimental lignin condensation reactions.

In this study, we evaluated poplar solvolysis as a function of temperature, residence time, and solvent system and measured the rate and extent of lignin extraction in an FT reactor.^15^ Through characterization of the extracted lignin molar mass distribution, β-*O*-4 content, and hydrogenolysis monomer yield, we quantified the lignin condensation during extraction. From this, we demonstrated that higher temperatures increased the lignin extraction extent, reaching a maximum at 250 °C of 60 and 93 wt % for methanol and 1:1 v/v methanol–water, respectively. Condensation reactions for both solvent systems were more impacted by residence time, with greater extraction and condensation occurring in methanol–water compared to pure methanol for all temperatures and residence times. This work highlights the benefits of water as a cosolvent for maximizing lignin extraction and emphasizes the need for precise control of reactor residence time necessary to limit condensation reactions in FT-RCF.

## Materials and Methods

To evaluate the impact of process conditions on lignin extraction and condensation extents, FT-solvolysis reactions were performed on duplicate 5 g beds of hybrid poplar (26.4 wt % lignin), first with methanol as the extraction solvent. During FT-solvolysis, the solvent was pumped into a heated reactor containing a fixed bed of poplar to extract lignin. The stream was then quickly cooled to ambient temperature in a knockout pot with an external cooling coil to avoid further condensation. Solvolysis reactions were run at 175 °C, 200 °C, 225 °C, and 250 °C and residence times of 9-, 18-, and 36-min, corresponding to solvent flow rates of 4, 2, and 1 mL/min, respectively. The solvolysis reactions were all maintained at 1600 psig by a back pressure regulator downstream of the knockout pot to ensure the solvent remains in the condensed phase. The residence time was controlled by adjusting the flow rate of the pump while holding all other parameters constant.

Four main samples were produced and analyzed in each experiment, which are noted in italics in Figure 1. Residual biomass was recovered from the reactor after each experiment. After drying it was weighed and analyzed by compositional analysis.Time course solvolysis liquor samples were collected every 30 min, inclusive of a heat ramp during the first 30 min time-on-stream, for a total of 2 h. Analytical solvolysis liquor samples were produced by combining all time course solvolysis liquor samples and removing the solvent by rotary evaporation to produce a lignin oil solution at a final total volume of 100 mL containing 2.9–12.4 mg/mL of solubilized lignin. Hydrogenolysis liquor samples were then produced by running batch hydrogenolysis reactions on the analytical solvolysis liquor. Hydrogenolysis reactions were carried out in a 75 mL Parr reactor at 225 °C for 3 h with 150 mg of 5 wt % Ru/C, 30 bar H_2_ charged at room temperature, and 30 mL of analytical solvolysis liquor in duplicate for each solvolysis reaction.

**Figure 1:**
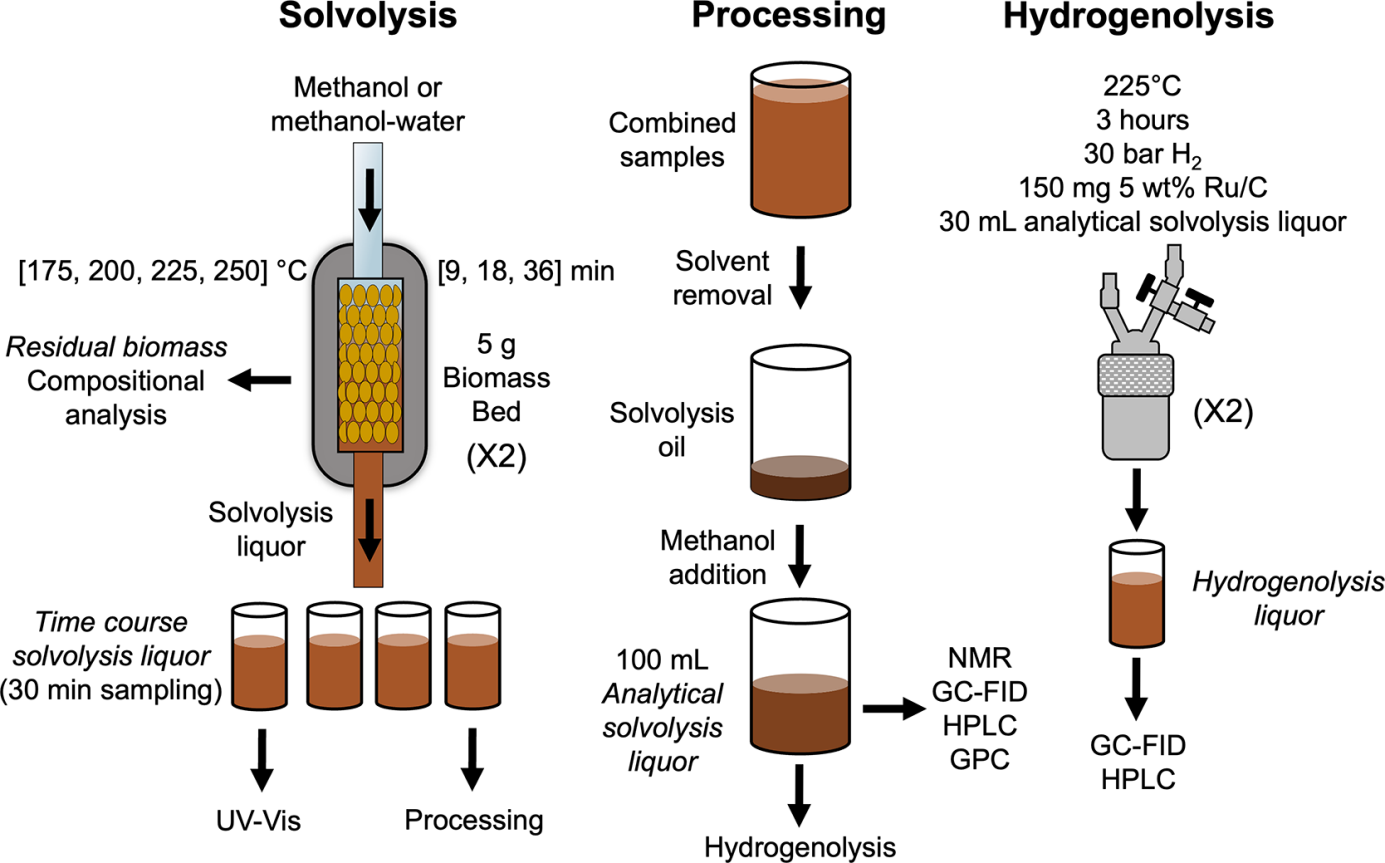
Schematic of the experimental procedure for the production and analysis of extracted lignin. Solvolysis reactions were conducted in duplicate with 5 g of hybrid poplar at 175 °C, 200 °C, 225 °C, and 250 °C for 9-, 18-, and 36-min residence times. Time course solvolysis liquor samples were collected every 30 min over 2 h of time-on-stream (inclusive of a 30 min heat ramp). UV–visible (UV–vis) spectroscopy measurements were taken on each of the individual time course solvolysis liquor samples. These samples were then combined to produce an aggregate solvolysis liquor sample. The solvent was removed to produce a normalized aggregate sample at 100 mL total volume of analytical solvolysis liquor. NMR, gas chromatography with flame ionization detection (GC-FID), high-performance liquid chromatography (HPLC), and gel permeation chromatography (GPC) were conducted on the analytical solvolysis liquor. Duplicate hydrogenolysis reactions were run for 3 h at 225 °C with 150 mg 5 wt % Ru/C, 30 bar H_2_, and 30 mL analytical solvolysis liquor to produce hydrogenolysis liquor with phenolic monomer quantification by GC-FID and HPLC.^43^

We used multiple metrics to compare the effects of reaction conditions on the delignification and condensation of lignin in FT solvolysis. Total delignification was used to quantify the amount of lignin extracted from the biomass based on recovered mass and compositional analysis (eq 1).

1 $$total\;delignification=\left(\frac{M_{BLi}-M_{BLf}}{M_{BLi}}\right)\times 100\%$$

Equation 1: total delignification is the difference in mass of lignin in the biomass, *M*_BL_, initial (i) and final (f) after 2 h time-on-stream (TOS) solvolysis reactions, divided by the total lignin in the original biomass.

Delignification rate quantifies the lignin extraction as a function of time based on lignin measurements from the time course solvolysis liquor (eq 2).

$$delignification\;rate=\frac{\left(\Sigma M_{Lt2}-{\Sigma M}_{Lt1}\right)/\left(M_{BLi}\right)}{{TOS}_{t2}-{TOS}_{t1}}$$ 2

Equation 2: delignification rate is the difference in cumulative mass of extracted lignin, *M*_L_, divided by the mass of lignin in the biomass, *M*_BLi_, all divided by the specified TOS interval. Subscripts indicate the time point, *t*1, in terms of TOS, and results are reported as wt %/h. This metric represents the fraction of the total lignin extraction that would occur in 1 h at the rate measured between TOS *t*2 and *t*1.

Solvolysis monomer yield quantifies the amount of phenolic lignin monomers liberated during solvolysis that remain stabilized in the solvolysis liquor (eq 3).

3 $$solvolysis\;monomer\;yield=\frac{M_{S}}{M_{BLi}-M_{BLf}}$$

Equation 3: Solvolysis monomer yield is the total mass of lignin monomers in the analytical solvolysis liquor, *M*_s_, divided by the mass of extracted lignin, initial (i) minus final (f) lignin in the biomass, *M*_BL_.

Hydrogenolysis monomer yield was used to quantify the amount of lignin produced by complete hydrogenolysis of the analytical solvolysis liquor (eq 4). This excludes the solvolysis monomer yield.

4 $$hydrogenolysis\;monomer\;yield=\frac{M_{H}}{M_{BLi}-M_{BLf}}\times 100\%$$

Equation 4: Hydrogenolysis monomer yield is the mass of monomers produced by hydrogenolysis of the analytical solvolysis liquor (exclusive of solvolysis monomers *M*_s_), *M*_H_, divided by the mass of extracted lignin, initial (i) minus final (f) lignin in the biomass, *M*_BL_.

The extent of condensation was calculated as the ratio of monomers resulting from hydrogenolysis reactions to the “theoretical” monomer yield measured in batch RCF reactions. This was used as an indirect measurement of total condensation (eq 5).

5 $$Extent\;of\;condensation=\left(1-\frac{M_{H}}{M_{RCF}}\right)\times 100\%$$

Equation 5: The extent of condensation is a percentage equal to one minus the ratio of monomers produced by hydrogenolysis, *M*_H_, divided by the theoretical monomer yield measured from batch RCF reactions, *M*_RCF_.

The initial and final lignin content was measured by compositional analysis using the recovered biomass after each solvolysis experiment.^44^ Extracted lignin in TOS samples was analyzed by UV–vis spectroscopy utilizing Beer’s law and calculating the molar absorptivity, ε, based on the total delignification measured by compositional analysis (Figure 2, see Supporting Information for details). Aliquots of analytical solvolysis liquor were analyzed by GC-FID for phenolic monomers and HPLC for quantification of p-hydroxybenzoic acid (pHBA), which cannot be quantified by GC-FID. Heteronuclear single quantum coherence nuclear magnetic resonance (HSQC NMR) spectroscopy was used to estimate the amount of β-*O*-4 linkages before and after hydrogenolysis reactions to verify that the selected hydrogenolysis conditions achieved complete aryl-ether bond cleavage. GPC was used to identify shifts in molar mass distribution. GC-FID and HPLC were used to quantify aromatic monomers in hydrogenolysis liquor.

**Figure 2:**
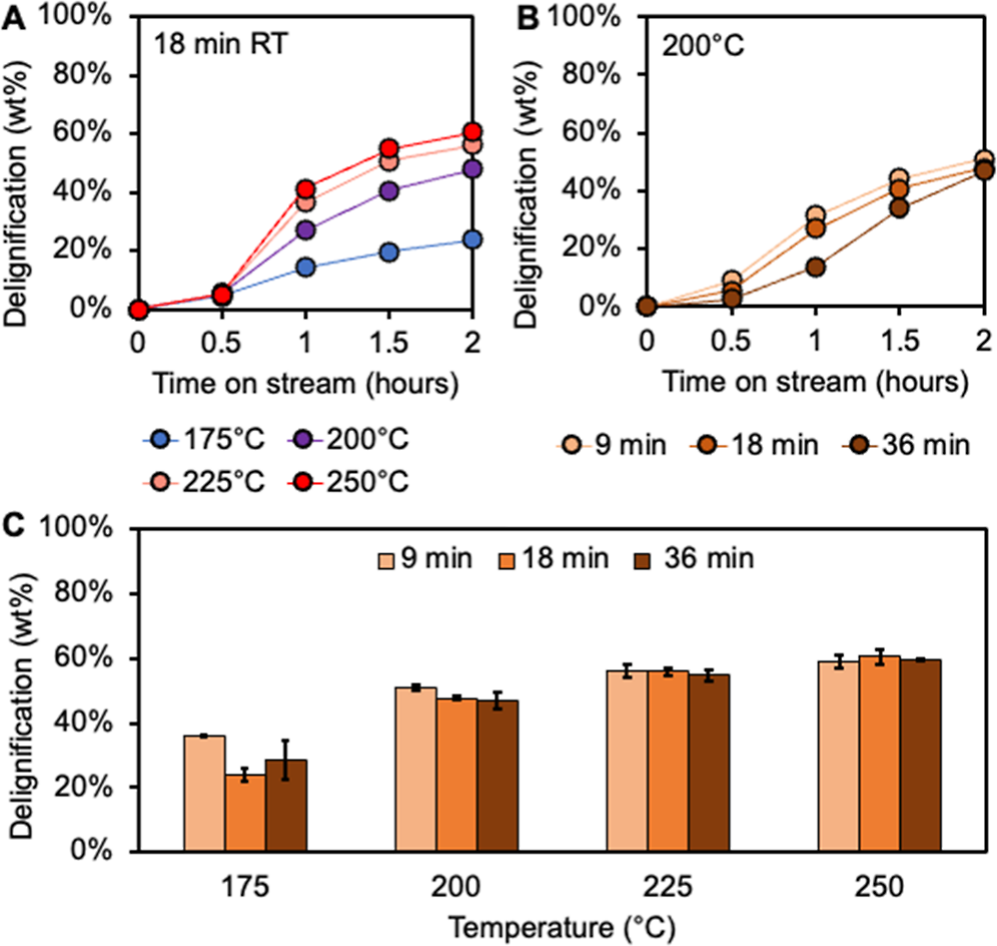
Delignification in FT-solvolysis with methanol. (A) Cumulative lignin extracted in time course solvolysis liquor for 18-min residence time at 175 °C, 200 °C, 225 °C, and 250 °C measured by UV–vis spectroscopy. (B) Cumulative lignin extracted in time course solvolysis liquor for 200 °C with 9-, 18-, and 36-min residence times measured by UV–vis spectroscopy. (C) Total delignification of biomass for three residence times (9-, 18-, and 36-min) at each temperature (175 °C, 200 °C, 225 °C, and 250 °C) measured by compositional analysis (Table S1).^44^ Solvolysis experiments were conducted with 5 g of poplar at the defined residence time and temperature. The first 30 min was a heat ramp to reach reaction temperature. Each experiment was conducted in duplicate. Error bars represent the range of duplicate results. Time course data for 9- and 36-min residence times are shown in Figures S1 and S2 and time course data for 175°, 225°, and 250 °C in Figures S3–S5. Quantitative results for these data are available in Supplemental Excel file 1.

## Results and Discussion

### Methanol Solvolysis

Lignin extraction has been hypothesized to occur via multiple distinct steps, including diffusion of solvent into the biomass, cleavage of lignin linkages, and diffusion of extracted lignin out of the biomass.^45^ Delignification results from FT-solvolysis experiments demonstrated a strong temperature dependence for both the rate and extent of lignin extraction (Figure 2A). Increasing temperature exhibited increasing delignification rates over 0.5 to 1 h TOS of 19.7, 43.3, 62.5, and 72.6 wt %/h for 18-min residence time solvolysis experiments at 175 °C, 200 °C, 225 °C, and 250 °C, respectively (Figure 2A). In all conditions, the maximum delignification rate occurs during 0.5 to 1.0 h time-on-stream when the reactor first reaches reaction temperature, similar to previous studies.^46^ Following this initial phase of rapid extraction, the rate of extraction slows over time as the concentration of available lignin remaining in the biomass decreases (additional time course data in Figures S1–S5). The total delignification after 2 h TOS shows a strong temperature dependence, with delignification only reaching 23.8 wt % at 175 °C but increasing to 60.4 wt % at 250 °C for the 18-min residence time. These results demonstrate that even for high temperature solvolysis with methanol, delignification appears to be limited to a maximum of ∼ 60–70 wt %.^15,47^

While the reaction temperature impacts both the rate and extent of delignification, the residence time impacts only the rate (Figure 2B). As expected, solvolysis experiments at 200 °C illustrate that increasing the residence time leads to a decreased delignification rate. During 0.5–1 h TOS, the delignification rate for the 9-min residence time was 44.7 wt %/h compared to 23.0 wt %/h, for 36-min residence time. Total delignification after 2 h TOS was nearly identical for all residence times at a fixed temperature (Figure 2C). These results indicate that increasing the residence time decreases the rate of lignin extraction, likely based on the differences in the lignin concentration gradient between the cell wall and solvent combined with higher extents of potentially condensed lignin redeposited onto the biomass. Higher residence times require a lower flow rate, which leads to a higher lignin concentration in solution based on equal extraction. Regardless of incremental extraction rates, the total delignification is determined by the reaction temperature.

Once extracted, lignin can undergo depolymerization through aryl-ether linkage cleavage to form reactive intermediates, which subsequently can undergo condensation reactions to form new C–C linkages. Together, these processes lead to a polymer that is recalcitrant to further upgrading and thus minimizing these deleterious reactions while simultaneously reaching high extents of extraction is an active area of interest.^7^ Previous studies have hypothesized that the solvent plays a critical role in depolymerizing lignin during extraction, acting both as a stabilizing agent via α-alkoxylation and as a hydrogen donor to facilitate the aryl ether cleavage.^34,37,48^

To investigate the condensation processes which occurred during FT-solvolysis, we first analyzed the extracted lignin structure via 2D ^1^H–^13^C HSQC NMR. Sels et al. hypothesized that lignin condensation occurs primarily through repolymerization of coniferyl and sinapyl alcohol-like intermediates derived from β-*O*-4 cleavage.^48^ The presence of β-*O*-4 linkages in the analytical solvolysis liquor (Figure 3C–F) was confirmed by the characteristic C_α_-H_α_ resonance at δ_C_/δ_H_ of 73.0/4.92 ppm.^48^ As the solvolysis temperature increases, β-*O*-4 abundance (expressed relative to total aromatic units calculated from *S*_2/6_ and *G*_2_ resonances) decreases. The maximum β-*O*-4 abundance of 46% was observed at 175 °C and 9-min residence time, while the minimum of 18% was measured for 250 °C and 36-min residence.^49^ Resonances attributed to coniferyl and sinapyl alcohol (C_α_–H_α_ δ_C_/δ_H_ ∼ 130/6.45 ppm, C_β_–H_β_ δ_C_/δ_H_ ∼ 128/6.20 ppm; Figure S6) were relatively constant at ∼8% across the studied temperature range, indicating that the higher rate of formation of these products via β-*O*-4 cleavage at increased temperatures is balanced by the rate of degradation by repolymerization (Figure S6I).^48^ Still, these data suggest that a substantial fraction of monomers from subsequent catalytic stabilization (vide infra) are derived from stabilization of coniferyl and sinapyl alcohol present in the analytical solvolysis liquor.^50^ The *S*/*G* ratio of the lignin also increased with temperature, which has also been noted by previous authors and hypothesized to arise from variation in where lignin resides in the plant cell wall. Lignin in the compound middle lamella, which is richer in guaiacyl lignin, is easier to extract compared to syringyl-rich lignin in the secondary cell wall.^51^

**Figure 3:**
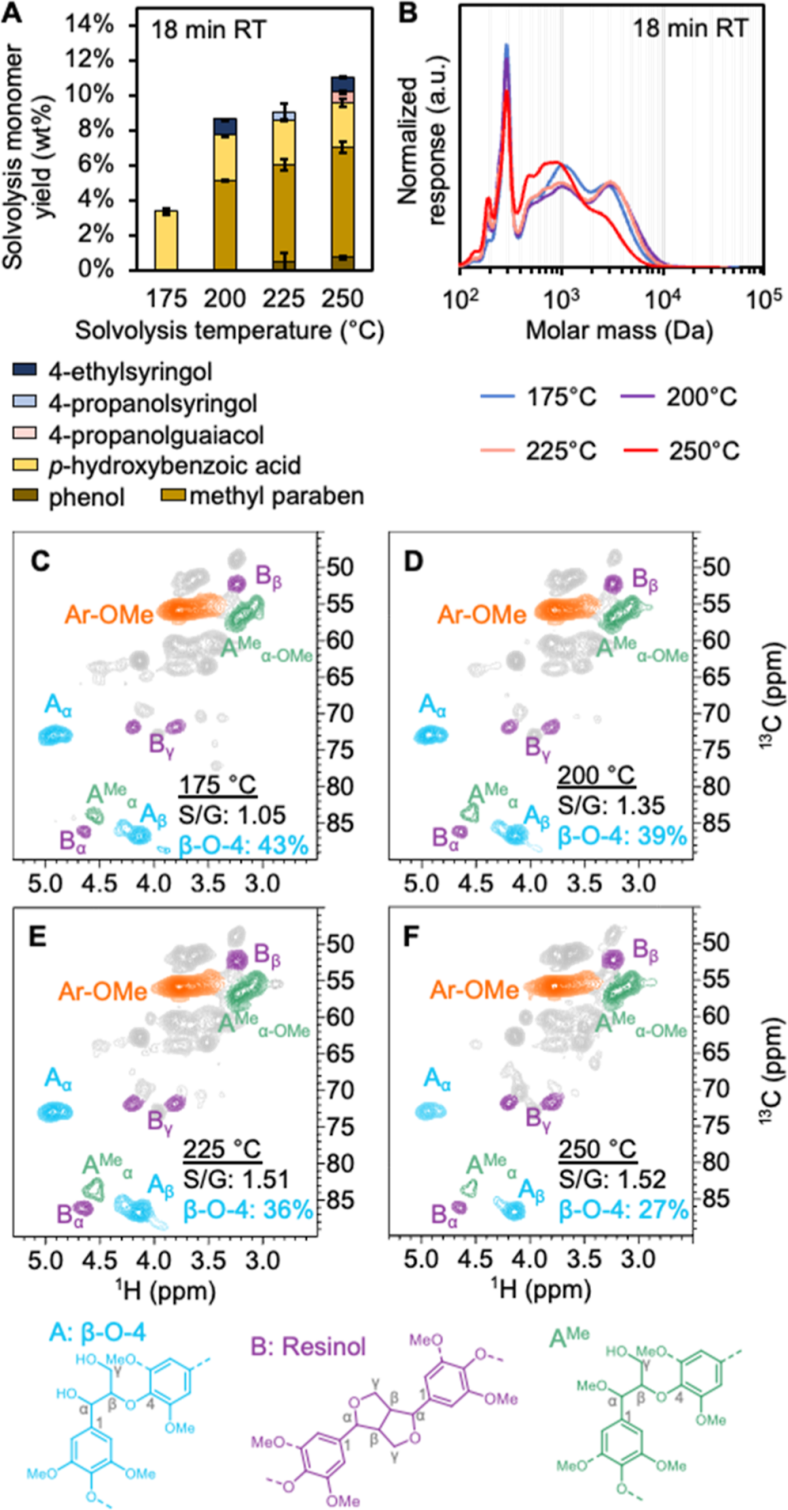
Methanol analytical solvolysis liquor results at 18-min residence time. (A) Solvolysis monomer yields measured by GC-FID and HPLC for 18-min residence time (see Figures S6 and S7, for 9- and 36-min solvolysis monomer yields). (B) GPC traces at 175 °C, 200 °C, 225 °C, and 250 °C for 18-min residence time (see Figure S8 and S9 for 9- and 36-min residence times). β-*O*-4 analysis by ^1^H–^13^C HSQC NMR at (C) 175 °C, (D) 200 °C, (E) 225 °C, and (F) 250 °C. Solvolysis reactions were run with 5 g of poplar at 18-min residence time with methanol as a solvent. Quantitative results for these data are available in Supplemental Excel file 1.

In addition to coniferyl and sinapyl alcohol, aromatic monomers, primarily in the form of *p*HBA and methyl paraben, were observed in the analytical solvolysis liquor. These samples showed that phenolic monomers constituted 3–12 wt % of the total extracted lignin at all temperatures and residence times (Figure 3A for 18-min residence time, Figures S7 and S8 for 9- and 36-min residence times). The dominant species was methylparaben at all temperatures except 175 °C, which produced only *p*HBA. Importantly, *p*HBA is known to be a pendant unit esterifying lignin γ-hydroxyl groups of syringol units.^52^ Its presence in solvolysis samples indicates the cleavage of these labile ester bonds rather than monomer formation from β-*O*-4 bond cleavage. The increasing *p*HBA and methylparaben contents of the extracted lignin with increasing temperature may be linked to the S/G ratio. The syringol units, which are esterified with *p*HBA, become more abundant later in the lignin extraction process. The presence of syringol and guaiacol compounds in the analytical solvolysis liquor at higher temperatures combined with the decreasing abundance of β-*O*-4 content with increasing temperature indicates that some β-*O*-4 cleavage occurs during solvolysis. However, the fact that all analytical solvolysis liquor samples contain some degree of β-*O*-4 content also indicates that the hydrogenolysis catalyst is crucial in cleaving the residual ether bonds left intact after solvolysis to liberate more monomeric and low molecular weight lignin. As discussed below, the complete absence of β-*O*-4 linkages in the hydrogenolysis liquor also corroborates this observation.

GPC showed that the molar mass distribution shifted from higher to lower molar mass components with increasing temperature (Figure 3B for 18-min residence time, Figures S9 and S10 for 9- and 36-min residence time), also suggesting a simultaneous increase in depolymerization reactions at higher temperatures.

### Hydrogenolysis after Methanol Solvolysis

After determining the amount of lignin extracted and aromatic monomers produced by solvolysis, we sought to quantify the degree of condensation that occurred during the solvolysis reactions. To this end, we first performed batch RCF reactions on hybrid poplar to determine the maximum monomer content achievable for the temperatures used for the solvolysis reactions. We then performed hydrogenolysis reactions on the analytical solvolysis liquor. The temperature, time, catalyst loading, and hydrogen pressure (225 °C, 3 h, 150 mg of 5 wt % Ru/C, and 30 bar H_2_) were selected to ensure complete cleavage of the aryl-ether linkages, which was verified by HSQC NMR (Figure S11). These yields, termed hydrogenolysis monomer yield, reflect the aromatic monomers derived from catalytic cleavage of β-*O*-4 linkages, as well as stabilization of coniferyl alcohol and sinapyl alcohol already present in the extracted lignin. In order to deconvolute the total delignification from the extent of condensation, we present hydrogenolysis monomer yield on the basis of total delignification rather than total lignin present in the initial biomass. For instance, when batch RCF was performed at 225 °C on hybrid poplar, the monomer yield on the basis of total lignin loaded, the traditional metric reported in RCF literature, was 23.6 wt %.^9^ The average total delignification at 225 °C was 55.6 wt % resulting in a hydrogenolysis monomer yield of 42.4 wt % (23.6%/55.6%). Utilizing the analytical solvolysis liquor generated at 225 °C with an 18-min residence time, the resulting monomer yield on the basis of total lignin loaded was 20.4 wt % with an average total delignification of 56.0 wt % and a corresponding hydrogenolysis monomer yield of 36.3 wt % (20.4%/56.0%). The lower monomer content in the hydrogenolysis liquor compared to the maximum content obtained in the batch RCF control experiments is attributed to the occurrence of condensation during solvolysis. Based on this metric, we express the extent of condensation that occurred during solvolysis as the ratio of hydrogenolysis monomer yield to the control batch RCF monomer yield.^9^ The 225 °C described above results in an extent of condensation of 14.4 wt % (1–36.3%/42.4%).

The hydrogenolysis monomer yields, total delignification, and extent of condensation for these reactions are shown in Figure 4. The batch RCF controls exhibited similar hydrogenolysis monomer yield across the temperature range from 175 to 250 °C, with a slight increase with increasing temperature, ranging from 38.3 to 51.2 wt %. The total delignification increases with increasing temperature but shows little dependence on the residence time. The average total delignification across 9-, 18-, and 36-min residence times for 175 °C, 200 °C, 225 °C, and 250 °C was 27.1, 48.5, 55.6, and 59.7 wt %, respectively. While higher temperatures resulted in higher total delignification, it also led to higher extents of condensation with temperatures of 175 °C, 200 °C, 225 °C, and 250 °C resulting in 29.4, 30.3, 38.1, and 70.8 wt %, respectively, for a 36-min residence time. The monomer yield for the 9-min residence time was generally comparable to the 18-min residence time at each temperature. Monomer selectivity remained consistent across temperatures and residence times (Figure S12).

**Figure 4:**
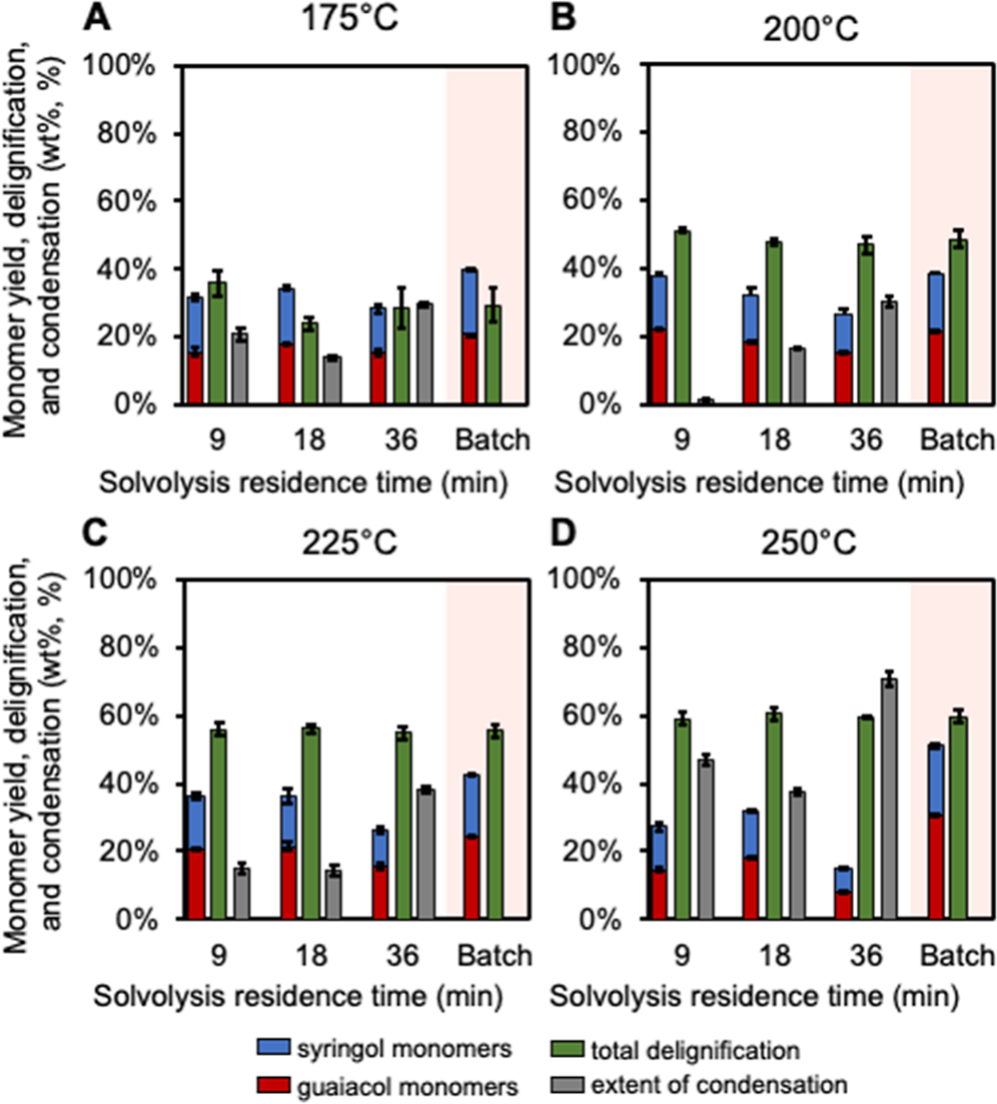
Comparison of hydrogenolysis monomer yields from methanol solvolysis to batch RCF in methanol. (A) 175 °C, (B) 200 °C, (C) 225 °C, and (D) 250 °C for 9-, 18-, and 36-min residence times. Monomer yields and delignification are measured as wt % and condensation is measured as %. Solvolysis reactions were run with 5 g of poplar at the defined residence time and temperature. “Guaiacol monomers” indicates a combined quantification of 4-substituted ethylguaiacol, propylguaiacol, isoeugenol, and propanolguaiacol. “Syringol monomers” is a combined quantification of 4-substituted ethylsyringol, propylsyringol, propenylsyringol, and propanolsyringol. All hydrogenolysis reactions were run in duplicate at 225 °C for 3 h with 150 mg of 5 wt % Ru/C and 30 bar H_2_ using 30 mL of analytical solvolysis liquor. Monomer yield error bars are the standard deviation of four hydrogenolysis reactions, two for each duplicate solvolysis reaction. Delignification and condensation error bars are the range of duplicate measurements. Quantitative results for these data are available in Supplemental Excel file 1.

Overall, these data demonstrate that condensation reactions occur rapidly during lignin extraction in methanol, and this increases with increasing temperature. Compared to the generally accepted mechanism of acid-catalyzed condensation,^23^ the mechanism of lignin condensation under near-neutral conditions is less understood.^35^ Previous reports have repeatedly shown that β-O-4 containing model compounds readily degrade in methanol at similar reaction temperatures, even without added acid.^34,53^ The major product of these reactions is coniferyl alcohol, which subsequently can be methylated at the gamma position. This methylation may temporarily protect these intermediates;^54^ however, uncatalyzed reactions using coniferyl alcohol as the starting material show low recovery of detectable products, indicating it is similarly susceptible to degradation.^48,50^ Furthermore, weak acids such as acetic acid can be formed from acetate groups on hemicellulose during extraction, which may accelerate this process.^55^

### Methanol–Water Solvolysis

Following the methanol solvent evaluation, we then evaluated a methanol–water solvent system. Water is a useful cosolvent in lignin-first reactions, as it can increase the lignin extraction extent relative to alcohol-only solvents from ∼60 wt % to >90 wt %.^11,39,47,56^ Water as a cosolvent will also decrease the reaction pressure, thus positively impacting capital costs.^10,57^ However, a potential trade-off is increased lignin condensation susceptibility with water present.^58^ Based on our results for the methanol system, we chose to investigate all four temperatures for an 18-min residence time and all residence times at 200 °C. We hypothesized that the total delignification would depend on temperature, and the extent of condensation would depend on residence time, predominantly for the methanol–water system, based on the trends observed for the pure methanol solvent.

Solvolysis reactions with methanol–water showed a substantial increase in total delignification with a maximum of 92.7 wt % at 250 °C (Figure 5A). The correlation of increasing temperature and increasing total lignin extracted followed the trend expected from the methanol case, with average total delignification of 53.2, 72.8, and 80.4 wt % for 175 °C, 200 °C, and 225 °C, respectively. Delignification rates also increased with increasing reaction temperature, with rates of 27.0, 41.1, 51.6, and 72.8 wt %/h for 175 °C, 200 °C, 225 °C, and 250 °C, respectively, at 18-min residence time for 0.5–1 h TOS. Unlike methanol solvolysis, methanol–water solvolysis showed a continued increase in extraction rate from 1 to 1.5 h TOS, with rates climbing to 43.7, 72.4, 71.0, and 78.5 wt %/h at each increasing temperature. It is worth noting that delignification does not fully plateau by 2 h TOS as it did for the methanol reactions, but we maintained 2 h TOS to define total delignification for consistency with the methanol case.

**Figure 5:**
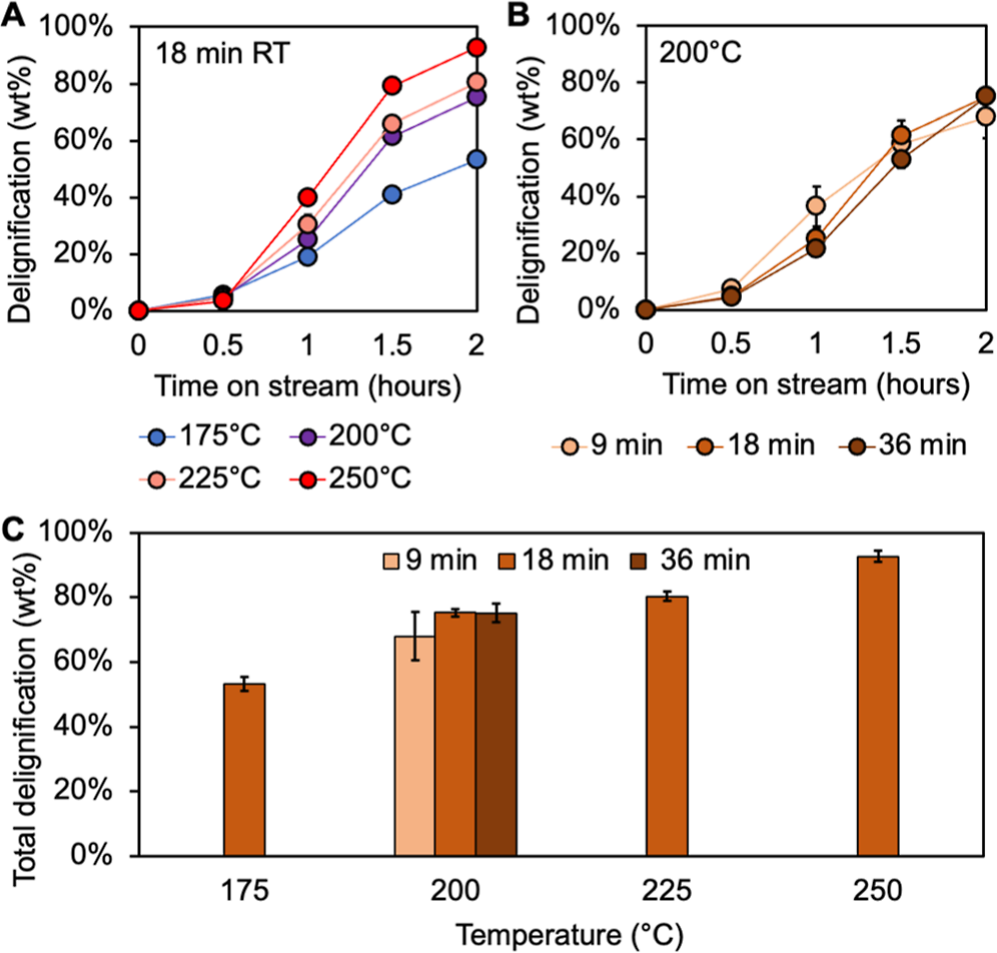
Delignification in FT-solvolysis with methanol–water. (A) Cumulative lignin extraction for time course lignin extraction for 18-min residence time at 175 °C, 200 °C, 225 °C, and 250 °C. (B) Cumulative lignin extraction for time course lignin extraction for 200 °C with 9-, 18-, and 36-min residence times. (C) Total delignification for three residence times (9-, 18-, and 36-min) at 200 °C and 18-min residence time at all other temperatures (175 °C, 225 °C, and 250 °C). Compositional analysis data are included in Table S2. Solvolysis experiments were conducted with 5 g of poplar at the defined residence time and temperature with 1:1 v/v methanol–water. The first 30 min was a heat ramp to reach reaction temperature. Each experiment was conducted in duplicate. Error bars represent the range of duplicate results. Quantitative results for these data are available in Supplemental Excel file 1.

Residence time effects for methanol–water were similar but not identical to the methanol experiments. Increasing the residence time for methanol–water solvolysis did not consistently decrease the rate of delignification as it did for methanol solvolysis. The delignification rate increased with decreasing residence time for 0.5–1 h TOS from 34.4, 41.1, and 58.3 wt %/h at 36-, 18-, and 9-min residence times and 200 °C (Figure 5B). The next 0.5 h TOS do not maintain this trend with corresponding rates of 63.1, 72.4, and 43.7 wt %/h, showing the lowest delignification rate for the shortest residence time. Previous reports have shown that water facilitates the extraction of hemicellulose in addition to lignin, which may result in increased pore sizes and faster diffusion of lignin from biomass.^40,56,59^ The trend of total delignification increasing with temperature and not depending on the residence time for the methanol case was also observed for methanol–water solvolysis (Figure 5C). The total delignification for methanol–water solvolysis increased by 39.5 wt % from 175 °C to 250 °C for 18-min residence times, but did not change with the three residence times at 200 °C, averaging 72.8 wt % with a standard deviation of 4.2 wt %.

Substantial differences are noticeable in the methanol–water solvolysis reactions compared to the methanol reactions from the analysis of the analytical solvolysis liquor. GC-FID and HPLC analysis from the samples showed a lower solvolysis monomer yield relative to total delignification, less than 8 wt % of the extracted lignin for all temperatures at an 18-min residence time, similar to that of the methanol case (Figures 6A and S13). The dominant species for the methanol–water system at all temperatures is *p*HBA. The solvolysis monomer yield with methanol–water decreased with increasing temperature, while methanol showed the inverse trend. The higher content of solvolysis monomers at lower temperatures may be due to water’s ability to facilitate lignin extraction from the secondary cell wall. ^1^H–^13^C HSQC NMR confirmed the presence of β–O–4 linkages in the solvolysis liquor at all temperatures tested (Figure 6C–F) and that the relative abundance of β–O–4 linkages continued to decrease with increasing solvolysis temperature, which is the same trend as the methanol results. Interestingly, coniferyl and sinapyl alcohol resonances were substantially lower compared to methanol solvolysis liquors, ranging from 0.6 to 3.2% for the methanol–water samples. This may indicate that the addition of water may prompt the repolymerization reactions of coniferyl and sinapyl alcohol. Additional resonances were also observed in the aliphatic region compared to solvolysis with methanol, which likely arises from the increase in hemicellulose solubilization.^49,60^ GPC analysis confirmed a decrease in monomers with increasing temperature, which is also the opposite trend of the GPC results from the methanol solvent system (Figures 6B and S14), suggesting more condensation reactions occurred at higher temperatures, as expected.

**Figure 6:**
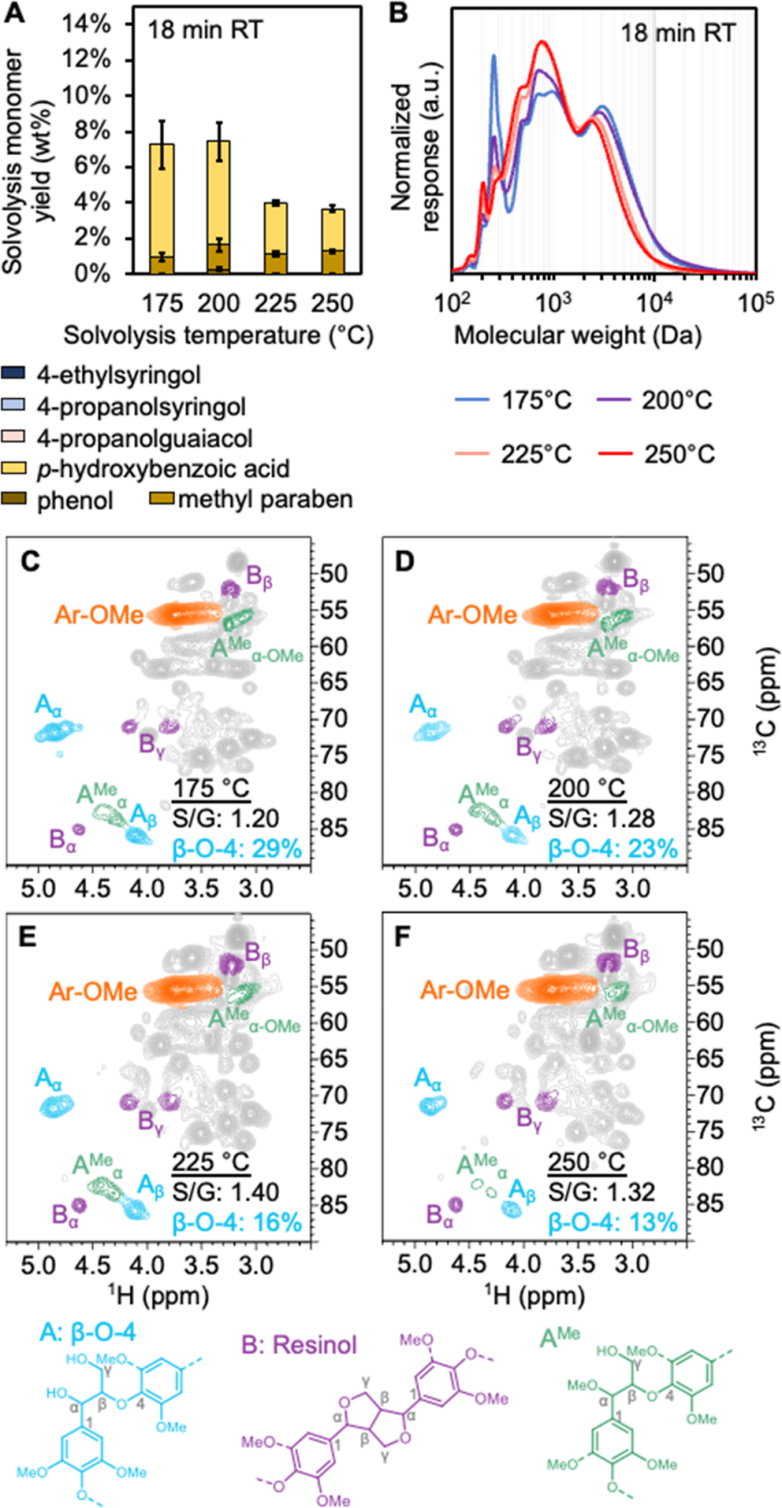
Methanol–water analytical solvolysis liquor results at 18-min residence time. (A) Solvolysis monomer yields for 18-min residence time and all temperatures (see Figure S13 for 200 °C and all residence times) and (B) GPC traces at 18-min residence time and all temperatures (see Figure S14 for 200 °C and all residence times). β-*O*-4 analysis by 2D HSQC NMR at (C) 175 °C, (D) 200 °C, (E) 225 °C, and (F) 250 °C. Solvolysis reactions were run with 5 g of poplar at 18-min residence time with 1:1 v/v methanol–water solvent. Note that the alpha methoxylated resonance (A_α‑OMe_) was not identified due to the presence of convoluting signals in the area. Quantitative results for these data are available in Supplemental Excel file 1.

### Hydrogenolysis of Methanol–Water Solvolysis

Hydrogenolysis reactions after methanol–water solvolysis were conducted in a manner identical to that for the methanol case to determine the availability of phenolic monomers from β-*O*-4 cleavage and catalytic stabilization to measure total condensed lignin. To isolate the impact of water as a cosolvent during solvolysis, the subsequent analytical solvolysis liquor was made by fully drying off the methanol–water solvent and dissolving the oil produced in pure methanol to a normalized volume of 100 mL for analysis and hydrogenolysis experiments. Control batch RCF experiments with poplar biomass were conducted in a 1:1 v/v methanol/water solvent mixture. The hydrogenolysis liquor was analyzed by ^1^H–^13^C HSQC NMR to confirm no β-*O*-4 remained after the reaction (Figure S15).

Batch RCF controls with methanol–water show a different response to increasing reaction temperature compared with methanol reactions. Hydrogenolysis monomer yield for methanol–water is shown in Figure 7 along with total delignification and extent of condensation. Increasing temperature for batch RCF controls results in a slight decrease of hydrogenolysis monomer yield with a maximum of 48.5 wt % at 175 °C and a minimum of 30.0 wt % at 250 °C. We observed increasing hydrogenolysis monomer yield with increasing temperature in methanol–water. These results indicate that the highest β-*O*-4 content is in the most labile lignin to extract, and that methanol alone is only able to extract this more labile lignin even at the maximum temperature of 250 °C.

**Figure 7:**
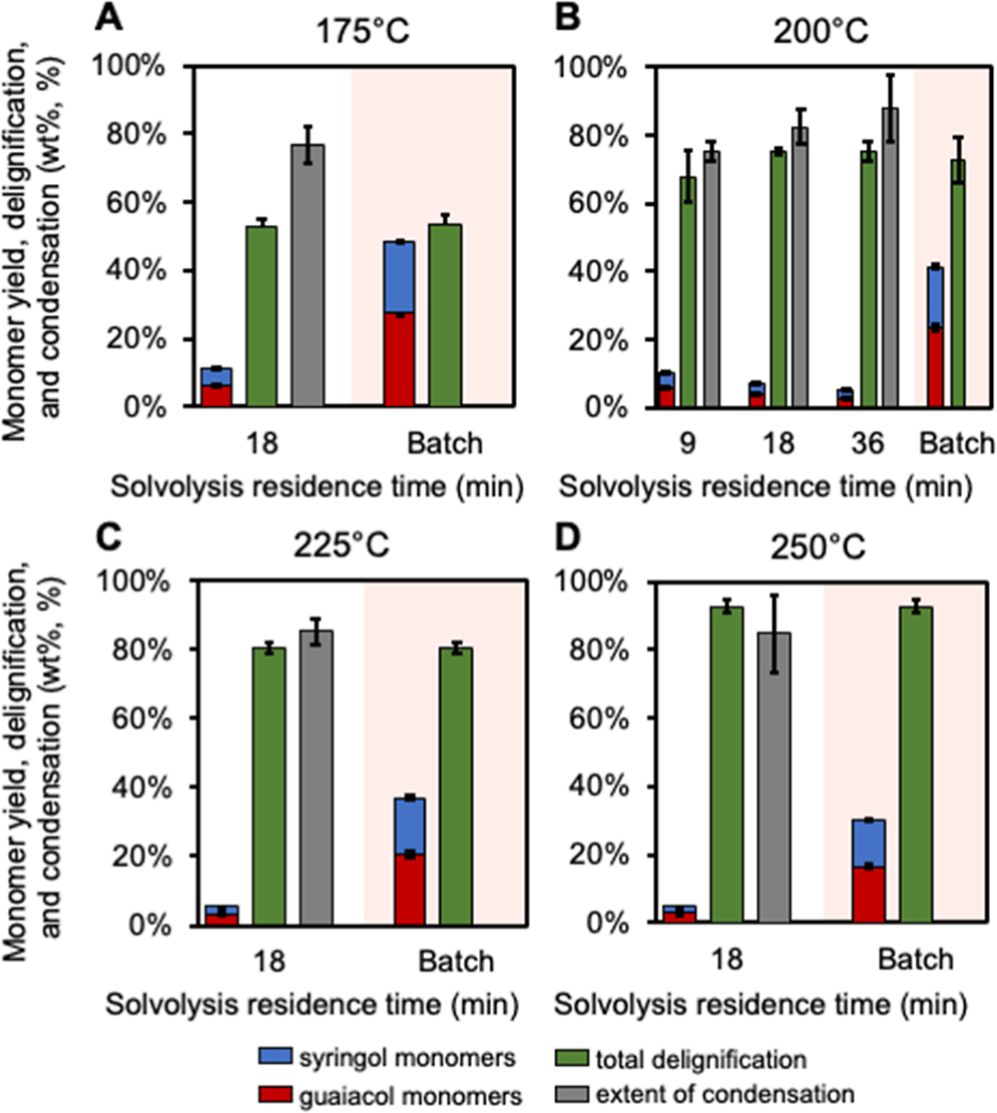
Comparison of hydrogenolysis monomer yields from methanol–water solvolysis to batch RCF in methanol=water. (A) 175 °C, (B) 200 °C, (C) 225 °C, and (D) 250 °C for 9-, 18-, and 36-min residence times. Monomer yields and delignification are measured as wt % and condensation is measured as %. Solvolysis reactions were run with 5 g of poplar at the defined residence time and temperature. “Guaiacol monomers” is a combined quantification of ethylguaiacol, propylguaiacol, isoeugenol, and propanolguaiacol. “Syringol monomers” is a combined quantification of ethylsyringol, propylsyringol, propenylsyringol, and propanolsyringol. All hydrogenolysis reactions were run in duplicate at 225 °C for 3 h with 150 mg of 5 wt % Ru/C and 30 bar H_2_ using 30 mL of analytical solvolysis liquor. Monomer yield error bars are the standard deviation of four hydrogenolysis reactions, two for each duplicate solvolysis reaction. Delignification and condensation error bars are the range of duplicate measurements. Quantitative results for these data are available in Supplementary Excel file 1.

The trends in the extent of condensation for methanol–water are the same as those observed for methanol. While acknowledging likely condensation reactions in the batch RCF control experiments, we still use the batch RCF reactions to determine the theoretical maximum monomer yield. The trend of increasing condensation with increasing residence time held for the 200 °C methanol–water case, where the extent of condensation was 75.1, 82.5, and 87.6 wt % for 9-, 18-, and 36-min residence times, respectively. The extent of condensation for the methanol–water system appears to be significant at all residence times and temperatures, with the lowest tested conditions being 200 °C and 9-min residence time, resulting in 75.1 wt % extent of condensation.

The effect of the addition of water during lignin extraction is likely multifaceted. Model compound experiments demonstrated that the rate of β-*O*-4 model compound cleavage and degradation was increased nearly 4× when water was used as the solvent for an uncatalyzed control experiment compared to ethanol.^61^ This strongly suggests that water prompts faster condensation compared to methanol without needing to consider other effects. Nonetheless, other phenomena may also contribute to the observed effect during lignin extraction with methanol-water mixtures. First, the increased extraction rate leads to an increase in lignin concentration in solution. Although the mechanism is uncertain, increasing the lignin concentration may increase the rate of condensation if the rate-limiting step is bimolecular, in which case the reaction order of lignin would be expected to be > 1. However, the lower sensitivity of condensation to residence time shown in this study may suggest that this effect is minor. Using water as the cosolvent also decreases the concentration of methanol in the reaction, which may decrease its ability to methoxylate the α position of β-*O*-4 moieties and/or the gamma position of reactive intermediates such as coniferyl alcohol, or may increase the rate of hydrolysis of the already methoxylated position. Increased hemicellulose extraction could lead to additional weak acids in solution (such as acetic acid), which could accelerate acid-catalyzed condensation as well.

## Conclusions

Independently controlling the solvolysis conditions for the extraction of lignin in flow revealed a strong temperature dependence for total delignification, with a maximum of 60.4 wt % for methanol occurring at 250 °C. The retention of aryl-ether linkages in the extracted lignin was more severely impacted by the residence time than by temperature, with the longest 36-min residence times showing the highest extents of condensation for all temperatures above 175 °C.

Adding water as a cosolvent significantly increased both the total delignification and the extent of condensation, resulting in a lower hydrogenolysis monomer yield as a weight percent of extracted lignin compared to methanol solvolysis for any tested temperature and 18-min residence time. Due to the higher rate of condensation in the methanol–water case, residence time had a more significant impact on monomer yield at 200 °C, with the short 9-min residence time showing a maximum hydrogenolysis monomer yield of 10.3 wt % with 18- and 36-min residence times decreasing to 7.3 and 5.1 wt %, respectively. These all exhibited significant condensation compared to the batch RCF reaction at 200 °C, which produced a 41.4 wt % hydrogenolysis monomer yield. Based on previous work by Jang et al., it would be expected that these trends would hold true for herbaceous and softwood feedstocks, but validating that assumption presents an opportunity for future work.^17^

These findings highlight the detrimental effect of water as a cosolvent in RCF reactions, leading to much higher condensation.^58^ Water improves the total lignin extraction but, due to increased condensation, results in lower monomer yields relative to the extracted lignin. Future opportunities for decreasing condensation while retaining the high delignification extent afforded by water as a cosolvent include further reducing the residence time between extraction and hydrogenolysis and selecting a more optimal cosolvent with water that could more effectively stabilize the extracted lignin. This study decouples the interplay of lignin extraction, condensation, and stabilization, which can inform the RCF solvent choice and reactor design.

## Supplementary Material





## Notes

Methods, figures referenced in main text, tabulated data for compositional analysis, and tabulated data for *S*/*G* ratios (PDF)
Data for all figures (XLSX)
