# Supplementary material for: Lignin Extraction and Condensation as a Function of Temperature, Residence Time, and Solvent System in Flow-through Reactors
Source: ACS Sustain Chem Eng. 2025 Aug 1;13(31):12573–82. doi: 10.1021/acssuschemeng.5c04198 (PMC12344771; doi:10.1021/acssuschemeng.5c04198)
Supplement: Supplementary file 1 [file sc5c04198_si_001.pdf]

# Supporting Information

**Lignin extraction and condensation as a function of temperature, residence time, and solvent system in flow-through reactors**

David G. Brandner,[1] Jaime Gracia Vitoria,[2] Jacob K. Kenny,[1] Jeremy Bussard,[1] Jun Hee Jang,[1] Sean P. Woodworth,[1] Karolien Vanbroekhoven,[2,*] Yuriy Roman-Leshkov,[3,*] and Gregg T. Beckham[1,*]

1. Renewable Resources and Enabling Sciences Center, National Renewable Energy Laboratory, Golden CO 80401 USA
2. Flemish Institute for Technological Research (Vito N.V.), Boeretang 200, Mol 2400, Belgium
3. Department of Chemical Engineering, Massachusetts Institute of Technology, Cambridge MA 02139 USA

* Correspondence: karolien.vanbroekhoven@vito.be; yroman@mit.edu; gregg.beckham@nrel.gov

Number of pages: 16
Number of figures: 16
Number of tables: 8

# 1. Supplemental methods

## 1.1 Reactor loading and operation

The reactor used in this work is originally described in our previous work.[1] Minor tubing modification were made that increased the residence time at 2 mL/min flow rate from 17 min to 18 min. For this study, the biomass beds for flow-through solvolysis consisted of ¾" Swagelok tubing with an inner diameter of 1.6 cm and isothermal length of 21.6 cm created by an oil jacket covered in custom heating elements from BriskHeat. Packing the bed consisted of loading a plug of quartz wool at the bottom of the bed, followed by inert silica chips, a second plug of quartz wool, 5 g of biomass, a third plug of quartz wool, inert silica chips, and finally a fourth plug of quartz wool. The inert silica packing was used to center the 10 cm plug of biomass in the isothermal zone.

After packing, the biomass beds were installed in the reactor utilizing VCR fittings and crush washers. They were pressure tested to 1800 psig with nitrogen then charged to 1600 psig with solvent at room temperature delivered by an HPLC pump. Once the reactor was filled and pressurized with solvent, heating and flow to the collection vessel were started. A thermocouple located inside the oil jacket indicated that the biomass bed reached reaction temperature in approximately 30 minutes for all temperatures. Samples were collected every 30 minutes after heating was initiated. During sampling, flow from the HPLC pump was paused, the collection vessel was isolated using a needle valve, then depressurized by venting the headspace to local exhaust, and then the sample was collected at ambient pressure. After the sample was collected the collection vessel was repressurized to 1600 psig with nitrogen utilizing an alternate flow path. Once the system was back at reaction pressure the HPLC pump was started again. Upon collection of the final sample all heating was turned off and the system was allowed to cool to room temperature. The solvent contained in the reactor during cooling was not collected or utilized in this work. Once the system was cool the biomass bed was removed, and the biomass was recovered. The biomass was then dried overnight in a hood prior to collecting a post-reaction mass. Biomass samples were then analyzed by the NREL LAP for compositional analysis.[2]

## 1.2 Materials

Methanol (≥99.9%, product number 34860) was purchased from Sigma Aldrich. Domestic water was purified by a Barnstead EASYpure II LF ultrapure water system (model number D7381). Ruthenium on carbon at 5 wt% loading was purchased from Sigma Aldrich (product number 206180). Hybrid poplar biomass was supplied by Idaho National Laboratory (INL) in 2021 after being harvested in Morrow County Oregon in 2013 by Greenwood Resources.

## 1.3 Compositional analysis

We refer the reader to the complete NREL LAP for compositional analysis.[2] Briefly, ash content was determined by placing an aliquot of the biomass sample in muffle furnace at 575°C until a constant weight was achieved. A separate aliquot was hydrolyzed using 72% sulfuric acid in a 30°C water bath for 1 hour. The hydrolyzed samples were then diluted and autoclaved at 121 °C for one hour. Acid-insoluble lignin was determined by filtering the hydrolysate through pre-ashed, pre-weighed crucibles, followed by drying at 105 °C and ashing at 575 °C to constant weight. All other measurements were collected using aliquots of the hydrolysate. Acid-soluble lignin was measured from using UV-Vis spectrophotometry within six hours of hydrolysis. Structural carbohydrates were quantified via HPLC using a Shodex sugar SP0810 column and refractive index detector following calcium carbonate neutralization and filtration of the hydrolysate. Acetyl content was determined using HPLC with an Aminex HPX-87H column and 0.005 M sulfuric acid as the mobile phase. Calibration standards and calibration verification standards (CVS) were used to ensure accuracy and reproducibility.

## 1.4 UV-Vis quantification of extracted lignin

One of the desired measurements from flow-through solvolysis experiments is the extent of delignification. A single solvolysis experiment produces four extracted lignin samples (taken every thirty minutes for two hours), and a single residual pulp. The measurement of lignin in solid biomass and residual cellulose rich materials is well established and can be readily applied to the residual pulp.[2] However, the measurement of solubilized lignin in each of the time course samples is less straightforward. In reductive catalytic fractionation experiments (RCF), a liquid-liquid extraction with an organic solvent (often either ethyl acetate or dichloromethane) and water is performed, and the mass of organic soluble oil is used to measure delignification. Lignin extracted in flow-through experiments is not extensively depolymerized, and it was expected that the liquid/liquid extraction would not sufficiently solubilize the lignin. Furthermore, while common amongst RCF practitioners, the delignification extent measured by organic soluble oil mass has also been shown to deviate from that measured by the established compositional analysis.[3] In place of this, extracted lignin in each time point sample was quantified with UV-Vis spectroscopy. UV-Vis has been used to quantify lignin in solution for various types of biomasses.[4] We acknowledge that the calculated lignin content is highly dependent on the absorptivity coefficient (ε) used. While the value of ε has been shown to vary for different types of biomass, it was expected that ε would be relatively consistent across the experiments (and within a single experiment) for a single type of poplar under similar conditions. Additionally, to more accurately reflect the lignin concentrations, the value of ε was adjusted for each experiment so that the delignification calculated from UV-Vis was equal to that measured from compositional analysis.

UV-Vis measurements were taken on a GENESYS™ 50 Vis/UV-Vis Spectrophotometer using a quartz cuvette. Total sample mass of each of the time-course extraction samples was measured by taking the mass of the sample vial before and after sample collection. An aliquot of this lignin solution was diluted 20x and 10x sequentially with methanol (200x total dilution) into a 4 mL sample vial using volumetric pipettes. Approximately 2 mL of this diluted solution was transferred the cuvette, and the UV-Vis measurement

was taken immediately. A scan of methanol was used as the background for all UV-Vis measurements. The absorbance at 240 nm was used for calculation of lignin concentrations in solution.

$$C = \frac{A_{240}}{\epsilon * l}$$

Where C is the lignin concentration in solution in g/L, $A_{240}$ is the absorbance value measured at 240 nm, ε is the absorptivity and l is the path length (1 cm).

As stated above, the absorptivity value was adjusted so that the calculated delignification from UV-Vis measurements was equal to the delignification measured by compositional analysis. The average calculated absorptivity across the samples was 34±5 L/(g·cm), which is similar those measured by Lavoie et al. for hemp and aspen at 237 nm.[4] See **Figure S16** for an example of how changing ε impacts the delignification profile.

## 1.5 Batch reductive catalytic fractionation (RCF)

Batch RCF was performed using 75 mL 5050 Parr reactors. 1.00 g of INL poplar biomass was loaded, along with 150 mg 5 wt% Ru/C catalyst and 23.76 g (30 mL) methanol or 27.79 g (30 mL) methanol-water into the reactors. The reactors where sealed, leak checked with 80 bar $N_2$, then flushed two more times with $N_2$ at 30 bar. Each reactor was charged with 30 bar $H_2$ and heated to reaction temperature over approximately 30 minutes. The reaction was carried out over three hours at temperature with stirring at 700 RPM. After 3 hours, the reactors were quenched in an ice water bath. Once cooled, samples were separated from the catalyst and biomass pulp using 0.2 μm filter.

## 1.6 Batch hydrogenolysis

The collected fractions from the flowthrough solvolysis experiments were combined into a single sample from each methanol and methanol-water run. For the methanol only experiments, A rotavapor (Buchi R-300) was used to reduce the total sample volume to approximately 100 mL or below and brought up with methanol to a total volume of 100 mL (79.20g). The Methanol/Water samples were dried down using a rotavapor (Buchi R-300) to an oil to completely remove the water fraction and reconstituted with 79.20 g (100 mL) methanol.

Batch hydrogenolysis was performed using 75 mL 5050 Parr reactors. 23.76 g (30 mL) of solvolysis liquor, along with 150 mg 5 wt% Ru/C catalyst were loaded into the reactors. The reactors where sealed, leak checked with 80 bar $N_2$, then flushed two more times with $N_2$ at 30 bar. Each reactor was charged with 30 bar $H_2$ and heated to 225 °C over approximately 30 minutes. The reaction was carried out over three hours at temperature with stirring at 700 RPM. After 3 hours, the reactors were quenched in an ice water bath. Once cooled, samples were separated from the catalyst using 0.2 μm filter.

## 1.7 GPC

*Gel Permeation Chromatography Analysis.* 15-20 mg of sample were acetylated using 0.5 mL pyridine (Sigma-Aldrich anhydrous 99.8%) and 0.5 mL of acetic anhydride (Sigma-Aldrich reagent plus ≥ 99%) sealed and heated to 40°C for 24 hours while stirring. Subsequently, 1 mL aliquots of methanol were then added to each sample and dried under $N_2$. This was repeated five times. Samples were then diluted in THF and stirred for 30 minutes. The THF solution was filtered through a 0.2 μm syringe filter into an HPLC vial. 20 μL of sample was injected on an HPLC fitted with three PLgel 7.5 x 300 mm columns in series: 10 μm x 50 Å, 10 μm x 103 Å, 10 μm x 104 Å (Agilent Technologies, Stockport, UK) at ambient temperature with an isocratic 1 mL $min^{-1}$ 100% tetrahydrofuran (Sigma-Aldrich inhibitor-free, suitable for HPLC ≥ 99.9%) for 40 minutes. Analytes are monitored at 210 nm, 260 nm, and 270 nm on the DAD.

## 1.8 2D $^1$H-$^{13}$C HSQC NMR

Samples were prepared for 2D $^1$H-$^{13}$C heteronuclear single quantum coherence (HSQC) experiments by transferring a 5-10 mL aliquot (the mass of the aliquot was measured gravimetrically) of the combined solvolysis liquors to a tared round bottom flask. The methanol solvent was evaporated in a rotary evaporator under reduced pressure and elevated temperature (~80 mbar, 35 °C) which yielded the crude lignin extract. The mass of the crude oil was measured gravimetrically. In an attempt to isolate the lignin, 5 mL of acetone was added to the crude lignin oil, and the round bottom was swirled to the dissolve the lignin. Upon addition, the liquid acetone phase was dark brown, and a white solid precipitate formed on the walls of the round bottom, presumably hemicellulose which had been co-extracted. The acetone was pipetted out of the round bottom and filtered through a 0.2 μm PTFE filter into a second tared round bottom. The acetone wash was repeated two additional times (total of three acetone washes). The acetone was then evaporated in a rotary evaporator which yielded a purified lignin oil and the mass was taken gravimetrically. The sample was dissolved in 1 mL of deuterated solvent. Acetone-$d_6$ was used as the solvent for methanol solvolysis samples and DMSO-$d_6$ was used for methanol/water solvolysis samples. Next, 0.5 mL of this sample was transferred to an NMR sample tube, along with 0.5 mL of a 2 g/L internal standard solution in (TTB/acetone-$d_6$ for MeOH solvolysis liquors; phloroglucinol/ DMSO-$d_6$ for MeOH/water solvolysis samples). Occasionally, the sample was cloudy precipitate indicating incomplete solubilization, in which case 0.25 mL of methanol- $d_4$ was added which fully resolubilized the NMR sample.

$^1H$ and $^1H$-$^{13}C$ HSQC NMR experiments were performed at 25°C on a Bruker Avance III 400 MHz spectrometer at 9.4 T using a Prodigy cryoprobe with 2048 points and a SW of 15 ppm in the F2 ($^1H$) dimension and 256 points and SW of 220ppm in the F1 ($^{13}C$) dimension using a standard phase sensitive, gradient selective pulse sequence (Bruker pulse sequence: hsqcetgpsp). Data was processed using MestreNova 14.1. Spectra were referenced using the residual solvent signal (acetone-$d_6$: $\delta_H/\delta_C$ = 2.05/29.92 ppm, DMSO- $d_6$: $\delta_H/\delta_C$ = 2.50/39.51 ppm). Apodization was performed using gaussian multiplication (25.9215 Hz) in the f2 dimension and sine-squared (90°) apodization in the f1 dimension. Baseline correction was performed using a 0$^{th}$ degree polynomial. Zero filling was performed to twice the original FID size (f2: 1024→2048 ; f1: 256→512).

NMR assignments were based on previous reports. The resonances of the alpha methoxylated β-O-4 structure were assigned from Chen et al. [1, 5-7]

## 2. Supplemental figures

### Methanol

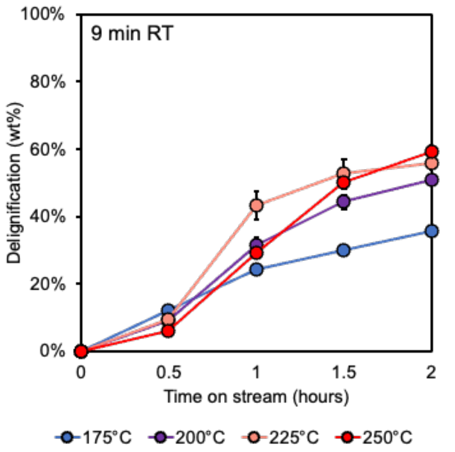


**Figure S1:** Cumulative lignin extracted timecourse for 9-min residence time comparing 175°C, 200°C, 225°C, and 250°C for pure methanol solvent as measured by UV-vis spectroscopy.

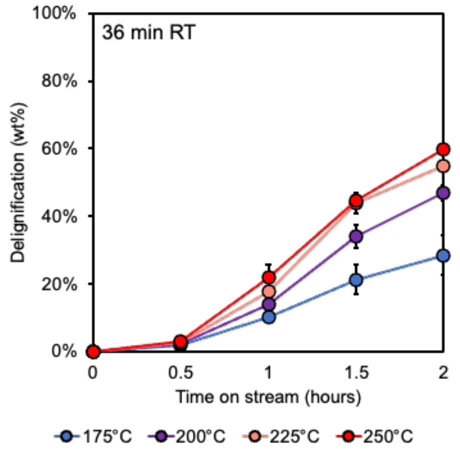


**Figure S2:** Cumulative lignin extracted timecourse for 36-min residence time comparing 175°C, 200°C, 225°C, and 250°C for pure methanol solvent as measured by UV-vis spectroscopy.

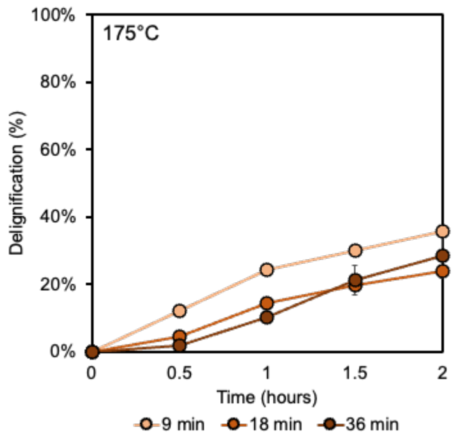


**Figure S3:** Cumulative lignin extracted timecourse for 175°C comparing 9-, 18-, and 36-minute residence time for pure methanol solvent measured by UV-vis spectroscopy.

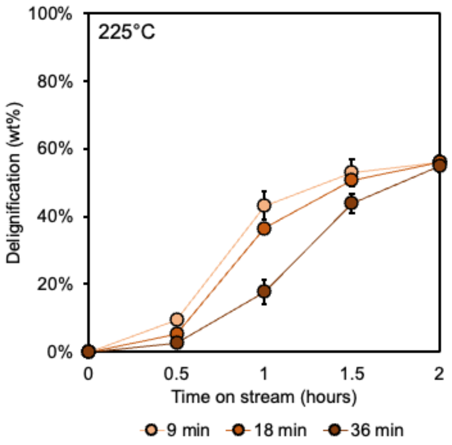


**Figure S4:** Cumulative lignin extracted timecourse for 225°C comparing 9-, 18-, and 36-minute residence time for pure methanol solvent measured by UV-vis spectroscopy.

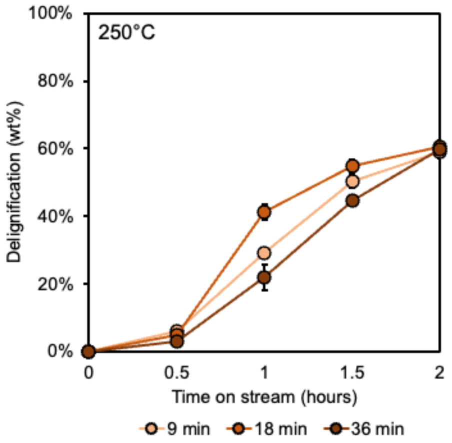


**Figure S5:** Cumulative lignin extracted timecourse for 250°C comparing 9-, 18-, and 36-minute residence time for pure methanol solvent measured by UV-vis spectroscopy.

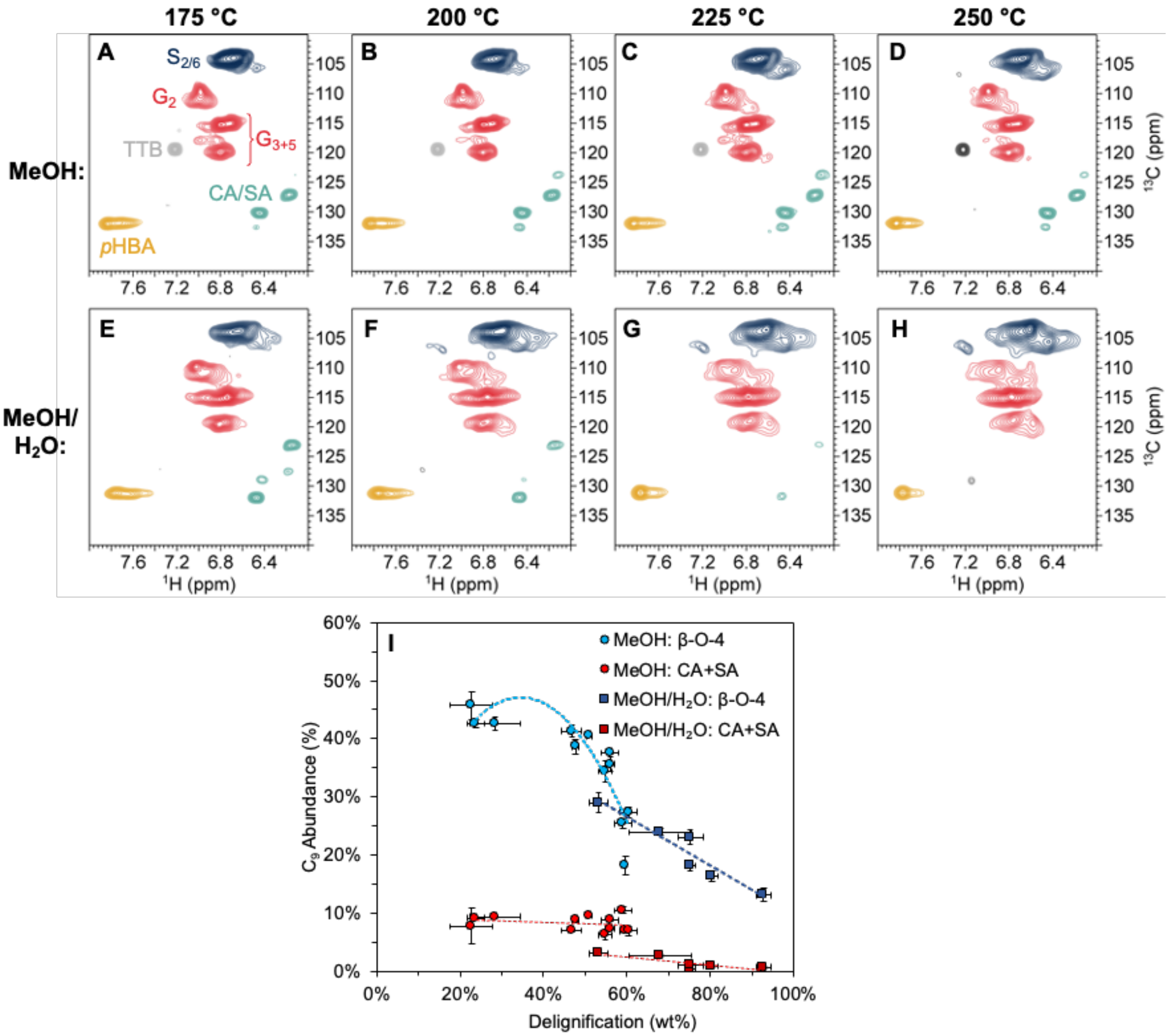


**Figure S6**. Resonances of coniferyl and sinapyl alcohol in the *analytical solvolysis liquor* for 18-minute residence time samples (**A-H**). Note that phloroglucinol was used as the internal standard for semi-quantitative analysis of the linkage abundance rather than TTB since TTB was not soluble in the DMSO-$d_6$ solvent needed to solubilize the solvolysis liquors from MeOH/$H_2O$ solvolysis. Quantification of the relative abundances of β-O-4 units and coniferyl/sinapyl alcohol resonances (**I**).

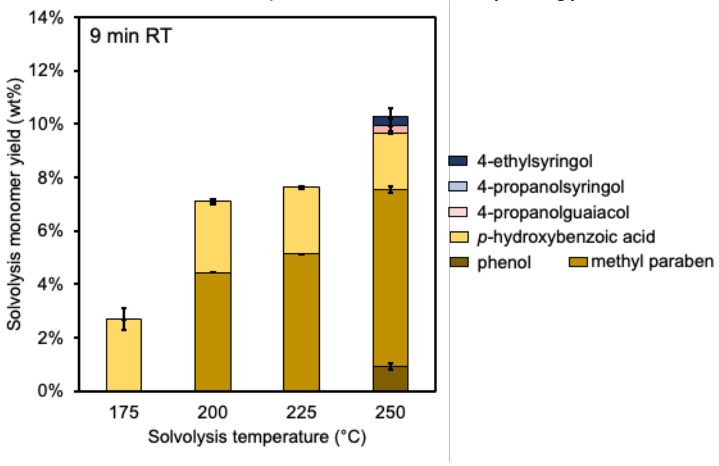


**Figure S7:** Solvolysis aromatic monomer yields for 9-minute residence time at 175, 200, 225, and 250°C.

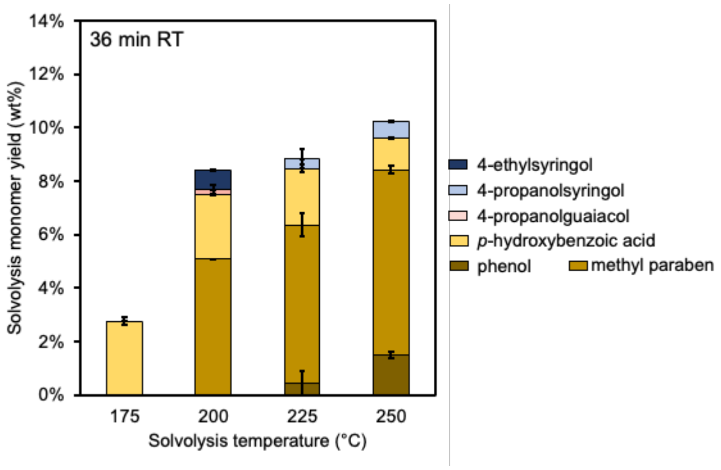


**Figure S8:** Solvolysis aromatic monomer yields for 36-minute residence time at 175, 200, 225, and 250°C.

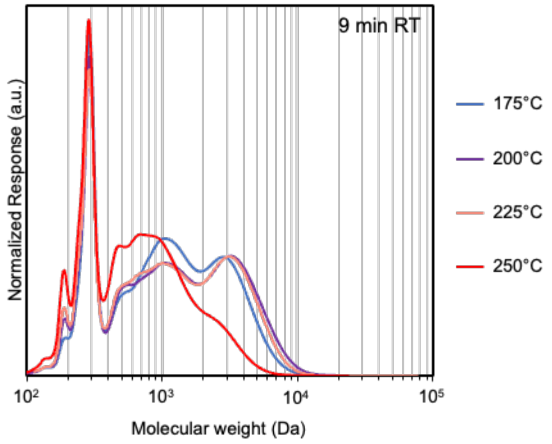


**Figure S9:** GPC of analytical solvolysis liquor for 9-minute residence time in pure methanol.

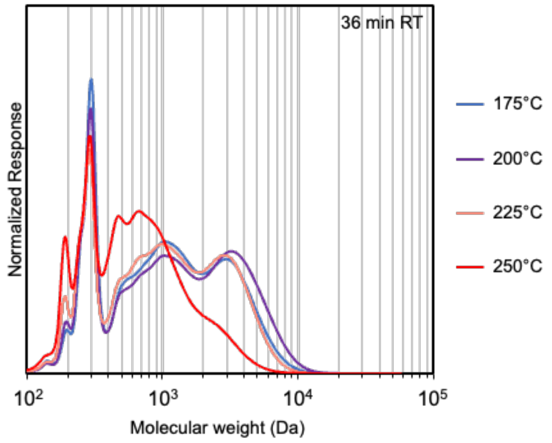


**Figure S10:** GPC of analytical solvolysis liquor for 36-minute residence time in pure methanol.

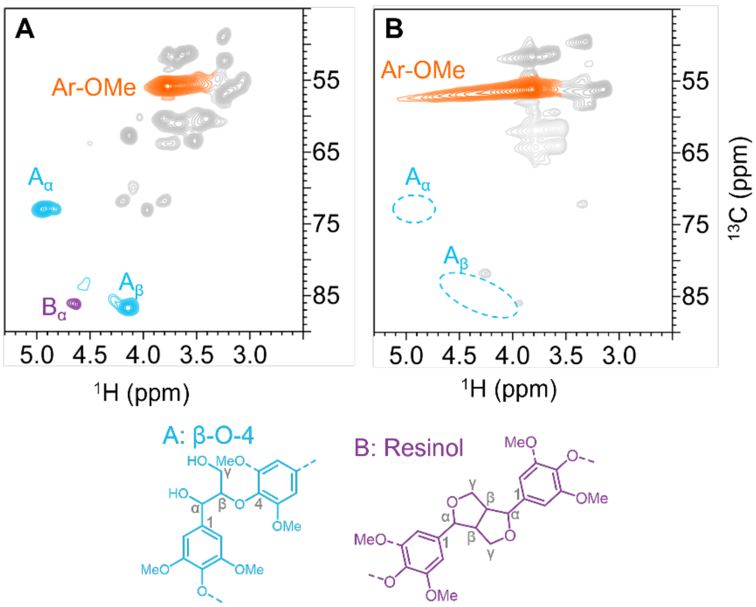


**Figure S11:** 2D $^{1}H$-$^{13}C$ HSQC representative solvolysis liquors (**A**) and hydrogenolysis liquors (**B**) for solvolysis reactions with methanol as the solvent. Solvolysis conditions: 5 g of poplar, 225 °C, 18-minute residence time. Hydrogenolysis conditions: 225 °C, 30 bar $H_2$, 3 hours, methanol, 150 mg of 5wt% Ru/C.

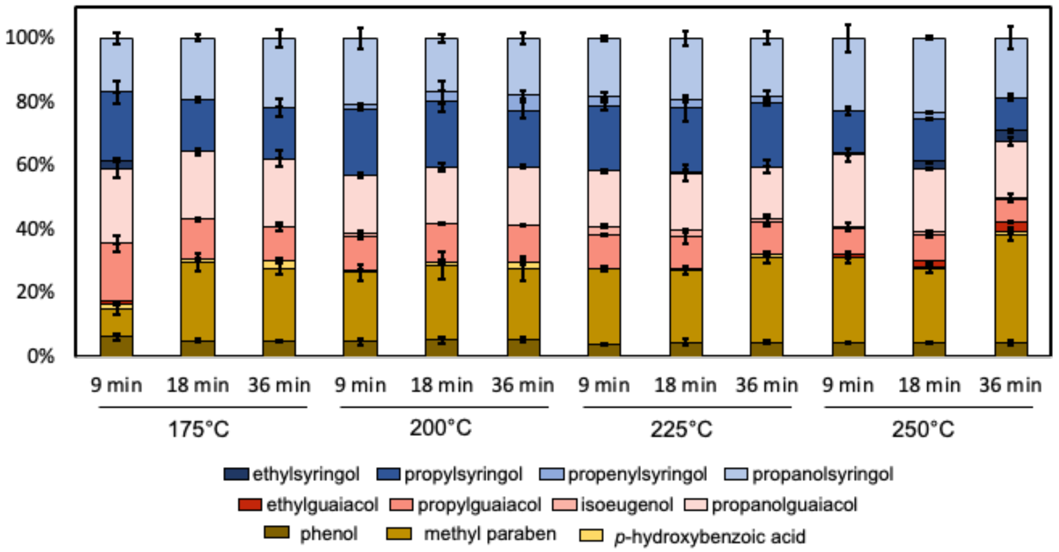


**Figure S12:** Hydrogenolysis liquor monomer selectivity for all solvolysis temperatures and residence times.

## Methanol-water

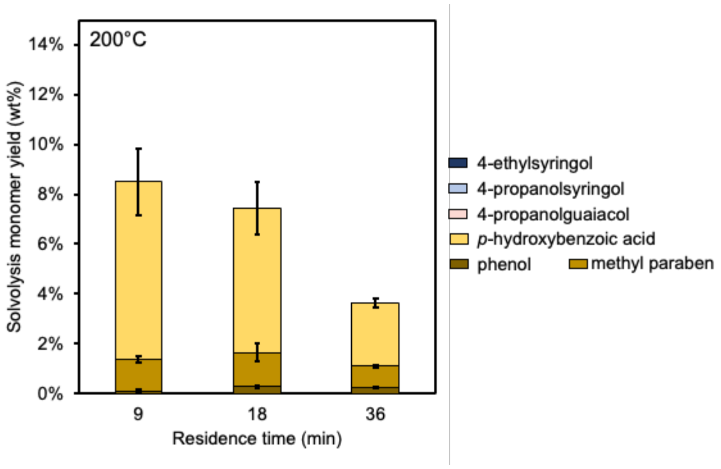


**Figure S13:** Solvolysis aromatic monomer yields at 200°C for 9-, 18-, and 36-minutes residence time in methanol-water.

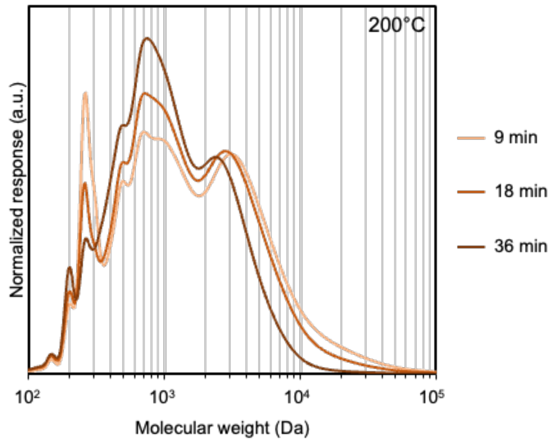


**Figure S14:** GPC of analytical solvolysis liquor at 200°C for 9-, 18-, 36-minutes in methanol-water.

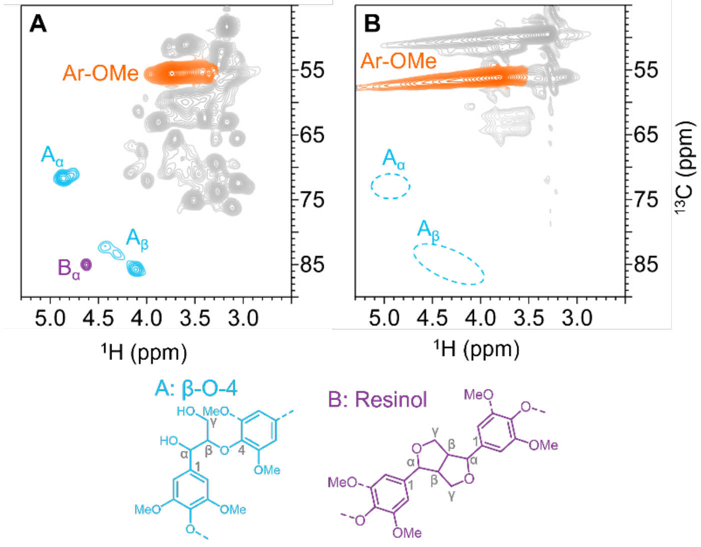


**Figure S15:** 2D $^{1}$H-$^{13}$C HSQC representative solvolysis liquors (**A**) and hydrogenolysis liquors (**B**) for solvolysis reactions with methanol-water as the solvent. Solvolysis conditions: 5 g of poplar, 225 °C, 18-minute residence time. Hydrogenolysis conditions: 225 °C, 30 bar $H_2$, 3 hours, methanol-water, 150 mg of 5wt% Ru/C.

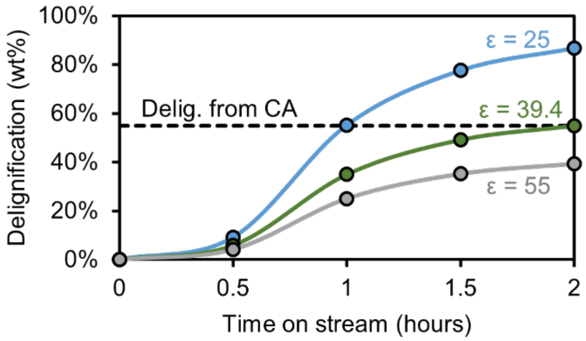


**Figure S16:** Impact of adjusting the absorptivity value, ε, on the delignification profile. For all delignification data, ε was adjusted so that the delignification value calculated from UV-Vis matched the delignification from compositional analysis (CA).

## 3. Supplemental data tables

**Table S1:** Compositional analysis for biomass residue for methanol flow-through solvolysis reactions. All values are weight % of sample.

| | | | | | Ash | Lignin | Glucan | Xylan | Galactan | Arabinan | Mannan | Acetyl | Total |
|---|---|---|---|---|---|---|---|---|---|---|---|---|---|
| 2021-INL Poplar | | | | | 0.64 | 26.37 | 44.88 | 12.84 | 1.33 | 0.22 | 2.76 | 3.49 | 96.30 |
| **Temp . (°C)** | **Residence time (min)** | **Rep .** | **Loaded biomass (wt%)** | **Recovered biomass (wt%)** | **Ash (wt%)** | **Lignin (wt%)** | **Glucan (wt%)** | **Xylan (wt%)** | **Galactan (wt%)** | **Arabinan (wt%)** | **Mannan (wt%)** | **Acetyl (wt%)** | **Total (wt%)** |
| 250 | 9 | 1 | 5.000 | 3.298 | 0.59 | 15.41 | 61.59 | 17.80 | 1.04 | 0.37 | 2.52 | 0.19 | 99.50 |
| | | 2 | 5.000 | 3.410 | 0.44 | 16.48 | 60.55 | 17.19 | 1.03 | 0.39 | 2.61 | 0.37 | 99.07 |
| | 18 | 1 | 5.005 | 3.010 | 0.60 | 16.30 | 62.57 | 17.68 | 1.00 | 0.38 | 1.87 | 0.05 | 100.45 |
| | | 2 | 5.008 | 3.260 | 0.54 | 16.78 | 62.36 | 17.67 | 0.99 | 0.39 | 2.09 | 0.11 | 100.93 |
| | 36 | 1 | 5.000 | 3.046 | 0.65 | 17.19 | 61.44 | 17.42 | 0.99 | 0.38 | 1.91 | 0.10 | 100.08 |
| | | 2 | 5.000 | 3.058 | 0.65 | 17.38 | 61.05 | 17.37 | 0.98 | 0.36 | 2.03 | 0.15 | 99.97 |
| 225 | 9 | 1 | 5.002 | 3.450 | 0.92 | 15.97 | 60.51 | 17.47 | 1.08 | 0.40 | 2.72 | 0.26 | 99.33 |
| | | 2 | 5.006 | 3.540 | 0.52 | 17.02 | 60.13 | 17.17 | 1.09 | 0.41 | 2.76 | 0.36 | 99.47 |
| | 18 | 1 | 5.006 | 3.620 | 4.89 | 16.31 | 59.15 | 16.95 | 1.09 | 0.42 | 2.52 | 0.20 | 101.52 |
| | | 2 | 5.007 | 3.530 | 1.32 | 15.91 | 61.51 | 17.47 | 1.07 | 0.45 | 2.46 | 0.17 | 100.36 |
| | 36 | 1 | 5.005 | 3.550 | 0.50 | 17.30 | 61.22 | 17.13 | 1.08 | 0.00 | 2.77 | 0.19 | 100.19 |
| | | 2 | 5.004 | 3.430 | 0.87 | 16.59 | 61.58 | 17.34 | 1.08 | 0.44 | 2.36 | 0.15 | 100.41 |
| 200 | 9 | 1 | 5.007 | 3.540 | 0.39 | 18.40 | 57.93 | 16.35 | 1.11 | 0.46 | 2.89 | 0.57 | 98.09 |
| | | 2 | 5.008 | 3.630 | 0.35 | 17.45 | 59.39 | 16.48 | 1.08 | 0.50 | 2.65 | 0.40 | 98.30 |
| | 18 | 1 | 5.009 | 3.510 | 0.36 | 19.23 | 57.99 | 16.40 | 1.10 | 0.49 | 2.72 | 0.44 | 98.73 |
| | | 2 | 5.000 | 3.600 | 0.49 | 19.31 | 57.63 | 16.56 | 1.12 | 0.51 | 2.73 | 0.46 | 98.80 |
| | 36 | 1 | 5.001 | 3.650 | 0.44 | 19.92 | 56.97 | 16.51 | 1.08 | 0.55 | 2.56 | 0.42 | 98.45 |
| | | 2 | 5.002 | 3.490 | 0.48 | 19.04 | 59.56 | 16.47 | 1.06 | 0.53 | 2.36 | 0.26 | 99.75 |
| 175 | 9 | 1 | 4.991 | 3.375 | 1.31 | 24.73 | 51.52 | 14.91 | 1.30 | 0.60 | 2.90 | 2.80 | 100.07 |
| | | 2 | 5.038 | 3.737 | 7.70 | 22.71 | 50.21 | 14.29 | 1.26 | 0.62 | 2.76 | 2.64 | 102.20 |
| | 18 | 1 | 5.004 | 4.150 | 0.37 | 24.68 | 51.60 | 14.72 | 1.31 | 3.10 | 3.41 | 1.92 | 101.12 |
| | | 2 | 5.008 | 4.070 | 0.42 | 23.90 | 51.67 | 14.56 | 1.34 | 3.05 | 3.37 | 2.00 | 100.32 |
| | 36 | 1 | 5.008 | 3.870 | 0.38 | 22.20 | 54.43 | 15.46 | 1.16 | 2.88 | 3.21 | 0.76 | 100.49 |
| | | 2 | 5.006 | 4.220 | 0.45 | 24.06 | 51.99 | 14.34 | 1.27 | 2.96 | 3.34 | 1.73 | 100.15 |

**Table S2:** Compositional analysis for biomass residue for methanol-water flow-through solvolysis reactions. All values are weight % of sample.

| Temp. (°C) | Residence time (min) | Rep. | Loaded biomass (wt%) | Recovered biomass (wt%) | Ash (wt%) | Lignin (wt%) | Glucan (wt%) | Xylan (wt%) | Galactan (wt%) | Arabinan (wt%) | Mannan (wt%) | Acetyl (wt%) | Total (wt%) |
|---|---|---|---|---|---|---|---|---|---|---|---|---|---|
| 250 | 18 | 1 | 5.000 | 2.046 | 2.86 | 3.51 | 90.22 | 2.71 | 0.40 | 0.00 | 2.14 | 0.00 | 101.85 |
| | | 2 | 5.001 | 2.159 | 0.89 | 5.44 | 89.90 | 3.11 | 0.00 | 0.00 | 2.47 | 0.00 | 101.81 |
| 225 | 18 | 1 | 5.001 | 2.680 | 0.63 | 8.87 | 83.14 | 6.55 | 0.00 | 0.00 | 3.75 | 0.49 | 103.44 |
| | | 2 | 5.002 | 2.769 | 1.24 | 9.94 | 79.20 | 7.09 | 0.00 | 0.00 | 3.62 | 0.70 | 101.80 |
| 200 | 9 | 1 | 5.003 | 2.796 | 1.16 | 11.46 | 77.24 | 7.40 | 0.00 | 0.00 | 3.80 | 1.02 | 102.08 |
| | | 2 | 5.000 | 3.180 | 1.12 | 16.29 | 66.78 | 10.60 | 0.00 | 0.00 | 3.93 | 1.97 | 100.68 |
| | 18 | 1 | 5.000 | 2.936 | 6.42 | 11.51 | 73.10 | 7.73 | 0.00 | 0.00 | 2.42 | 0.86 | 102.05 |
| | | 2 | 5.000 | 2.824 | 0.29 | 10.97 | 78.55 | 7.67 | 0.00 | 0.00 | 2.54 | 1.00 | 101.02 |
| | 36 | 1 | 5.001 | 2.832 | 5.53 | 10.06 | 74.29 | 6.72 | 0.00 | 0.00 | 2.37 | 0.65 | 99.63 |
| | | 2 | 5.002 | 2.598 | 0.89 | 13.98 | 73.83 | 8.78 | 0.00 | 0.00 | 2.59 | 1.21 | 101.29 |
| 175 | 18 | 1 | 5.002 | 3.347 | 0.18 | 17.44 | 66.52 | 10.92 | 0.00 | 0.00 | 2.71 | 2.27 | 100.04 |
| | | 2 | 5.003 | 3.520 | 0.26 | 18.25 | 64.01 | 11.63 | 0.00 | 0.00 | 2.70 | 2.39 | 99.23 |

**Table S3**. Hydrogenolysis monomer yields based on total lignin content for methanol solvolysis.

| Temp. (°C) | Residence time (min) | Syringol (wt%) | Stdev (wt%) | Guaiacol (wt%) | Stdev (wt%) | Total (wt%) | Stdev (wt%) |
|---|---|---|---|---|---|---|---|
| 250 | 9 | 8.6 | 0.9 | 7.5 | 0.6 | 16.1 | 1.1 |
| | 18 | 11.1 | 0.5 | 8.3 | 0.4 | 19.4 | 0.6 |
| | 36 | 4.8 | 0.3 | 4.2 | 0.2 | 8.9 | 0.4 |
| | Batch | 18.3 | 1.0 | 12.3 | 0.7 | 30.6 | 1.2 |
| 225 | 9 | 11.7 | 0.9 | 8.5 | 0.5 | 20.2 | 1.0 |
| | 18 | 11.9 | 1.5 | 8.4 | 0.9 | 20.4 | 1.7 |
| | 36 | 8.5 | 0.7 | 5.9 | 0.6 | 14.4 | 0.9 |
| | Batch | 13.7 | 0.6 | 9.9 | 0.4 | 23.6 | 0.8 |
| 200 | 9 | 11.3 | 0.6 | 7.9 | 0.4 | 19.2 | 0.7 |
| | 18 | 8.8 | 1.2 | 6.4 | 0.3 | 15.3 | 1.2 |
| | 36 | 7.2 | 0.9 | 5.3 | 0.3 | 12.5 | 0.9 |
| | Batch | 10.4 | 0.7 | 8.1 | 0.5 | 18.6 | 0.8 |
| 175 | 9 | 4.9 | 1.0 | 5.1 | 0.9 | 10.0 | 1.3 |
| | 18 | 4.2 | 0.5 | 4.0 | 0.4 | 8.2 | 0.6 |
| | 36 | 4.3 | 1.3 | 3.7 | 1.0 | 8.0 | 1.6 |
| | Batch | 6.1 | 1.1 | 5.6 | 1.0 | 11.7 | 1.5 |

**Table S4**. Hydrogenolysis monomer yields based on total lignin content for methanol-water solvolysis.

| Temp. (°C) | Residence time (min) | Syringol (wt%) | Stdev (wt%) | Guaiacol (wt%) | Stdev (wt%) | Total (wt%) | Stdev (wt%) |
|---|---|---|---|---|---|---|---|
| 250 | 18 | 2.5 | 0.7 | 1.7 | 0.3 | 4.4 | 0.7 |
| | Batch | 15.3 | 0.6 | 12.5 | 0.4 | 27.9 | 0.7 |
| 225 | 18 | 2.5 | 0.2 | 1.9 | 0.1 | 4.4 | 0.2 |
| | Batch | 16.5 | 0.9 | 13.3 | 0.9 | 29.8 | 1.3 |
| 200 | 9 | 4.0 | 0.7 | 3.0 | 0.6 | 7.0 | 0.9 |
| | 18 | 3.1 | 0.2 | 2.4 | 0.1 | 5.5 | 0.2 |
| | 36 | 2.2 | 0.4 | 1.6 | 0.2 | 3.9 | 0.4 |
| | Batch | 17.4 | 2.2 | 12.8 | 1.7 | 30.1 | 2.8 |
| 175 | 18 | 3.2 | 0.3 | 2.7 | 0.3 | 5.9 | 0.4 |
| | Batch | 14.5 | 0.9 | 11.2 | 0.8 | 25.8 | 1.2 |

**Table S5**. HSQC quantification of β-O-4, sinapyl and coniferyl alcohol, and S/G ratio for methanol solvolysis liquor samples. Percentages are expressed relative to the sum of the $S_{2/6} + G_2$ integrals.

| Temperature (°C) | Residence Time (min) | β-O-4 (%) | ± | SA+CA (%) | ± | S/G Ratio | ± |
|---|---|---|---|---|---|---|---|
| 250 | 9 | 25.4% | 1.2% | 10.6% | 0.8% | 1.51 | 0.03 |
| | 18 | 27.4% | 1.3% | 7.0% | 1.0% | 1.52 | 0.05 |
| | 36 | 18.3% | 2.4% | 7.1% | 0.1% | 1.58 | 0.00 |
| 225 | 9 | 37.6% | 0.3% | 8.9% | 0.3% | 1.47 | 0.04 |
| | 18 | 35.6% | 1.8% | 7.4% | 0.2% | 1.51 | 0.06 |
| | 36 | 34.4% | 2.7% | 6.3% | 1.1% | 1.49 | 0.00 |
| 200 | 9 | 40.5% | 0.0% | 9.6% | 0.2% | 1.42 | 0.07 |
| | 18 | 38.6% | 1.8% | 8.8% | 0.6% | 1.35 | 0.03 |
| | 36 | 41.3% | 1.5% | 7.1% | 0.4% | 1.41 | 0.03 |
| 175 | 9 | 45.7% | 3.3% | 7.8% | 4.4% | 1.08 | 0.14 |
| | 18 | 42.6% | 1.0% | 9.2% | 0.2% | 1.05 | 0.04 |
| | 36 | 42.7% | 1.6% | 9.4% | 0.1% | 1.09 | 0.11 |

**Table S6**. HSQC quantification of β-O-4, sinapyl and coniferyl alcohol, and S/G ratio for methanol-water solvolysis liquor samples. Percentages are expressed relative to the sum of the $S_{2/6} + G_2$ integrals.

| Temperature (°C) | Residence Time (min) | β-O-4 (%) | ± | SA+CA (%) | ± | S/G Ratio | ± |
|---|---|---|---|---|---|---|---|
| 250 | 18 | 13.1% | 1.6% | 0.8% | 0.3% | 1.32 | 0.02 |
| 225 | 18 | 16.5% | 1.2% | 0.9% | 0.1% | 1.40 | 0.11 |
| 200 | 9 | 24.0% | 0.8% | 2.8% | 0.3% | 1.20 | 0.07 |
| | 18 | 23.1% | 1.7% | 1.1% | 0.0% | 1.29 | 0.04 |
| | 36 | 18.3% | 1.2% | 0.6% | 0.2% | 1.32 | 0.01 |
| 175 | 18 | 29.0% | 2.4% | 3.2% | 0.4% | 1.20 | 0.04 |

**Table S7:** S/G ratio of hydrogenolysis monomers from methanol solvolysis.

| Temperature (°C) | Residence Time (min) | S/G |
|---|---|---|
| 250 | 9 | 1.14 |
| | 18 | 1.34 |
| | 36 | 1.14 |
| | Batch | 1.48 |
| 225 | 9 | 1.37 |
| | 18 | 1.42 |
| | 36 | 1.46 |
| | Batch | 1.38 |
| 200 | 9 | 1.42 |
| | 18 | 1.36 |
| | 36 | 1.34 |
| | Batch | 1.28 |
| 175 | 9 | 0.96 |
| | 18 | 1.07 |
| | 36 | 1.18 |
| | Batch | 1.08 |

**Table S8:** S/G ratio of hydrogenolysis monomers from methanol-water solvolysis.

| Temperature (°C) | Residence time (min) | S/G |
|---|---|---|
| 250 | 18 | 1.45 |
| | Batch | 1.23 |
| 225 | 18 | 1.30 |
| | Batch | 1.24 |
| 200 | 9 | 1.30 |
| | 18 | 1.28 |
| | 36 | 1.40 |
| | Batch | 1.36 |
| 175 | 18 | 1.18 |
| | Batch | 1.29 |

**References:**

(1) Brandner, D. G.; Kruger, J. S.; Thornburg, N. E.; Facas, G. G.; Kenny, J. K.; Dreiling, R. J.; Morais, A. R. C.; Renders, T.; Cleveland, N. S.; Happs, R. M. Flow-through solvolysis enables production of native-like lignin from biomass. *Green Chemistry* **2021**, *23* (15), 5437-5441.

(2) Sluiter, A.; Hames, B.; Ruiz, R.; Scarlata, C.; Sluiter, J.; Templeton, D.; Crocker, D. Determination of structural carbohydrates and lignin in biomass. *Laboratory analytical procedure* **2008**, *1617* (1), 1-16.

(3) Jang, J. H.; Morais, A. R. C.; Browning, M.; Brandner, D. G.; Kenny, J. K.; Stanley, L. M.; Happs, R. M.; Kovvali, A. S.; Cutler, J. I.; Román-Leshkov, Y.; et al. Feedstock-agnostic reductive catalytic fractionation in alcohol and alcohol–water mixtures. *Green Chemistry* **2023**, *25* (9), 3660-3670.

(4) Lee, R. A.; Bédard, C.; Berberi, V.; Beauchet, R.; Lavoie, J.-M. UV–Vis as quantification tool for solubilized lignin following a single-shot steam process. *Bioresource Technology* **2013**, *144*, 658-663.

(5) Talebi Amiri, M.; Bertella, S.; Questell-Santiago, Y. M.; Luterbacher, J. S. Establishing lignin structure-upgradeability relationships using quantitative 1H–13C heteronuclear single quantum coherence nuclear magnetic resonance (HSQC-NMR) spectroscopy. *Chemical Science* **2019**, *10* (35), 8135-8142.

(6) Li, Y.; Demir, B.; Ramos, L. M. V.; Chen, M.; Dumesic, J. A.; Ralph, J. Kinetic and mechanistic insights into hydrogenolysis of lignin to monomers in a continuous flow reactor. *Green Chemistry* **2019**, *21* (13), 3561-3572.

(7) Chen, J.; Lu, F.; Si, X.; Nie, X.; Chen, J.; Lu, R.; Xu, J. High yield production of natural phenolic alcohols from woody biomass using a nickel-based catalyst. *ChemSusChem* **2016**, *9* (23), 3353-3360.
